# Increasing the resilience of plant immunity to a warming climate

**DOI:** 10.1038/s41586-022-04902-y

**Published:** 2022-06-29

**Authors:** Jong Hum Kim, Christian Danve M. Castroverde, Shuai Huang, Chao Li, Richard Hilleary, Adam Seroka, Reza Sohrabi, Diana Medina-Yerena, Bethany Huot, Jie Wang, Kinya Nomura, Sharon K. Marr, Mary C. Wildermuth, Tao Chen, John D. MacMicking, Sheng Yang He

**Affiliations:** 1 Department of Biology, Duke University, Durham, NC USA; 2 Howard Hughes Medical Institute, Duke University, Durham, NC USA; 3 Department of Energy Plant Research Laboratory, Michigan State University, East Lansing, MI USA; 4 Plant Resilience Institute, Michigan State University, East Lansing, MI USA; 5 Department of Biology, Wilfrid Laurier University, Waterloo, Ontario Canada; 6 Howard Hughes Medical Institute, Yale University, West Haven, CT USA; 7 Yale Systems Biology Institute, Yale University, West Haven, CT USA; 8 Departments of Immunobiology and Microbial Pathogenesis, Yale University School of Medicine, New Haven, CT USA; 9 State Key Laboratory of Agricultural Microbiology, Huazhong Agricultural University, Wuhan, China; 10 Department of Plant Biology, Michigan State University, East Lansing, MI USA; 11 Department of Plant and Microbial Biology, University of California Berkeley, Berkeley, CA USA

**Keywords:** Plant hormones, Plant immunity, Climate-change impacts, Biotic

## Abstract

Extreme weather conditions associated with climate change affect many aspects of plant and animal life, including the response to infectious diseases. Production of salicylic acid (SA), a central plant defence hormone^1–3^, is particularly vulnerable to suppression by short periods of hot weather above the normal plant growth temperature range via an unknown mechanism^4–7^. Here we show that suppression of SA production in *Arabidopsis thaliana* at 28 °C is independent of PHYTOCHROME B^8,9^ (phyB) and EARLY FLOWERING 3^10^ (ELF3), which regulate thermo-responsive plant growth and development. Instead, we found that formation of GUANYLATE BINDING PROTEIN-LIKE 3 (GBPL3) defence-activated biomolecular condensates^11^ (GDACs) was reduced at the higher growth temperature. The altered GDAC formation in vivo is linked to impaired recruitment of GBPL3 and SA-associated Mediator subunits to the promoters of *CBP60g* and *SARD1*, which encode master immune transcription factors. Unlike many other SA signalling components, including the SA receptor and biosynthetic genes, optimized *CBP60g* expression was sufficient to broadly restore SA production, basal immunity and effector-triggered immunity at the elevated growth temperature without significant growth trade-offs. CBP60g family transcription factors are widely conserved in plants^12^. These results have implications for safeguarding the plant immune system as well as understanding the concept of the plant–pathogen–environment disease triangle and the emergence of new disease epidemics in a warming climate.

## Abstract (web summary)

Suppression of salicylic acid production in *Arabidopsis* *thaliana* at high temperature is caused by decreased recruitment of GUANYLATE BINDING PROTEIN-LIKE 3 defence-associated condensates on promoter sites of master immune regulatory genes.

## Main

Previous studies have shown that basal^13,14^ and pathogen-induced^15–17^ SA production are negatively affected by higher temperatures within the optimal plant growth range^13,14^ or short periods of heat waves above the optimal range^15–17^. The temperature sensitivity appears to be unique to the SA pathway, as other stress hormone pathways, such as jasmonate and abscisic acid, are upregulated at higher temperature^15,18^. The mechanisms underlying selective suppression of the SA pathway during heat waves above the optimal temperature range is unclear and remains controversial^15,16^, leaving a significant gap in our understanding of how a warming climate with frequent and extreme heat waves would influence the effectiveness of the plant immune system. This knowledge gap presents a major obstacle to developing climate-resilient plants in which SA-mediated defences operate effectively, a key concern for future agricultural productivity, ecosystem preservation and the emergence of new plant disease pandemics^4,5,19,20^.

## Temperature vulnerability of the SA pathway

**Fig. 1:**
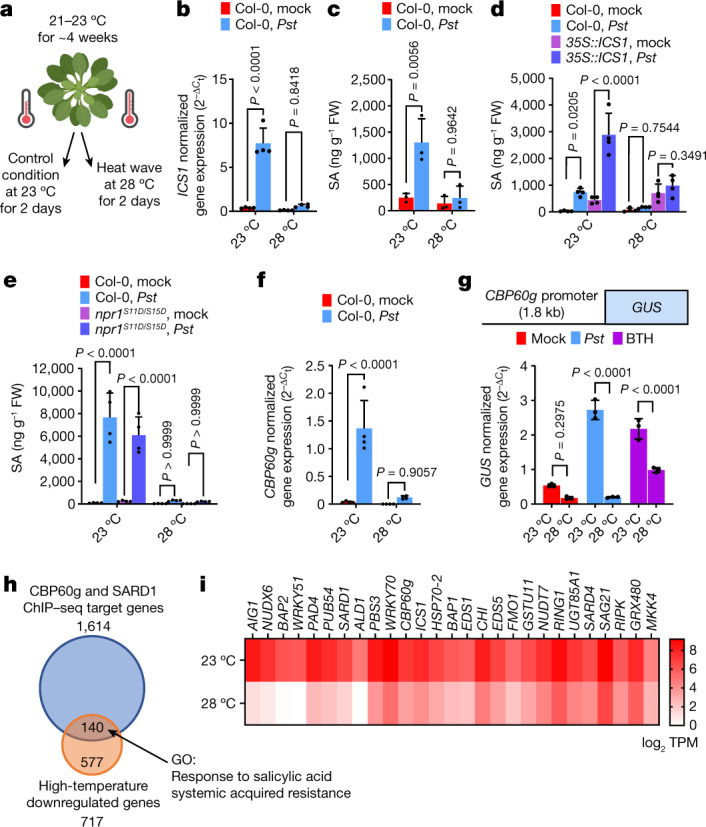
Temperature vulnerability of *CBP60g* gene expression and the SA transcriptome. Leaves of 4- to 5-week-old *Arabidopsis* plants were syringe-infiltrated with mock (0.25 mM MgCl_2_), *Pst* DC3000 (10^6^ colony forming units (CFU) per ml^−1^ suspension) or BTH solution and then incubated at 23 °C or 28 °C. Hormone analysis, RNA sequencing (RNA-seq), and quantitative PCR with reverse transcription (RT–qPCR) were performed 24 h after treatment (that is, 1 day post-inoculation (dpi)). **a**, A schematic diagram of the experimental protocol. **b**,**c**, *ICS1* transcript (**b**) and SA (**c**) levels in mock- and *Pst* DC3000-infiltrated Col-0 plants at 1 dpi. FW, fresh weight. **d**,**e**, SA levels in mock- and *Pst* DC3000-inoculated Col-0 (**d**,**e**) and *35S::ICS1* (**d**) or *npr1*^*S11D/S15D*^ (**e**) plants at 1 dpi. **f**, Endogenous *CBP60g* transcript level of samples in **b** at 1 dpi. **g**, Top, schematic of the *GUS* reporter gene. Bottom, *GUS* reporter gene expression in mock-, *Pst* DC3000- and BTH-treated *pCBP60g::GUS* plants one day after treatment. **h**, Gene Ontology (GO) analysis of *Pst* DC3000-induced genes that are differentially regulated at elevated temperature and their overlap with the SARD1 and CBP60g ChIP–sequencing dataset^31^. **i**, Representative RNA-seq reads after *Pst* DC3000 infection of defence-related CBP60g target genes for plants in **h**. TPM, transcripts per million mapped reads. Data in **b**–**g**,**i** are mean ± s.d. (*n* = 3 (**c**,**g**,**i**) or 4 (**b**,**d**–**f**) biological replicates) from one representative experiment analysed with two-way ANOVA with Tukey’s honest significant difference (HSD) for significance. Experiments were independently performed three times, except for **i**, with two experiments. Exact *P*-values for all comparisons are shown in the Source Data. Source data

**Extended Data Fig. 1:**
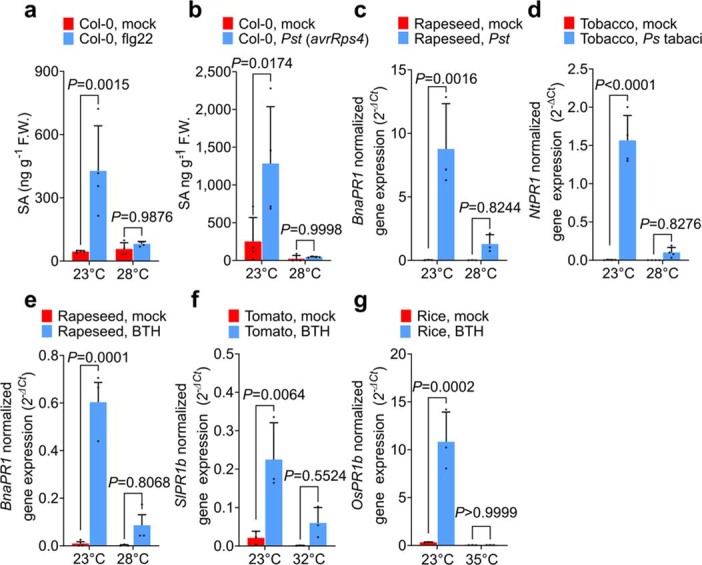
The SA pathway is downregulated at elevated temperatures in different plant species examined. **a–b**, SA levels in 4-week-old Col-0 plants at 24 h after treatment [i.e., 1 day post-inoculation (dpi)] with flg22 peptide treatment (**a**) or *Pst* DC3000(*avrRps4*) inoculation [1.0 x 10^8^ Colony Forming Units (CFU) mL^−1^] (**b**) at 23 °C and 28 °C. **c–d**, Transcript levels of *BnaPR1* in leaves of 4-week-old rapeseed Westar plants infiltrated with mock (0.25 mM MgCl_2_) or *Pst* DC3000 [1.0 x 10^5^ Colony Forming Units (CFU) mL^−1^] (**c**) and *NtPR1* in leaves of 4-week-old tobacco plants infiltrated with mock (0.25 mM MgCl_2_) or *Ps tabaci* 11528 [1.0 x 10^6^ Colony Forming Units (CFU) mL^−1^] (**d**) at 24 h post-inoculation (1 dpi) at 23 °C and 28 °C. **e**, *BnaPR1* expression levels in leaves of 4-week-old rapeseed Westar plants 1 day after mock (0.1% DMSO) or 50 µM BTH treatment at 23 °C and 28 °C **f**, SA marker gene (*SlPR1b*) expression levels in 4-week-old Castlemart tomato plants 1 day after mock (0.1% DMSO) or 100 µM BTH treatment at 23 °C and 32 °C. **g**, SA marker gene (*OsPR1b*) expression levels in 5-week-old rice plants 1 day after mock (0.1% DMSO) or 200 µM BTH treatment at 28 °C and 35 °C. Results show the means ± S.D. [n = 3 (**c**, **e**–**g**) or 4 (**a**, **b**, **d**) biological replicates] from one representative experiment (of three independent experiments) analyzed with two-way ANOVA with Tukey’s HSD for significance. Exact *P*-values for all comparisons are detailed in the Source Data files Source data

The model plant *A. thaliana* accession Col-0 becomes hypersusceptible to the virulent pathogen *Pseudomonas syringae* pv. *tomato* (*Pst*) DC3000 during a short period of growth at elevated temperature^15^ (Fig. 1a). Elevated temperature also suppressed the expression of *ISOCHORISMATE SYNTHASE 1*^15^ (*ICS1*) (Fig. 1b), a key SA biosynthetic gene^21^, leading to reduced SA accumulation at 28 °C versus 23 °C (Fig. 1c). Although elevated temperature does not affect MAP kinase activation during the early stages of pattern-triggered immunity (PTI) in response to bacterial flagellin-derived flg22 peptide^22^, downstream SA accumulation is significantly reduced (Extended Data Fig. 1a). Furthermore, consistent with previous studies showing suppressed effector-triggered immunity (ETI) at elevated temperature^22–25^, we found that SA accumulation in *Arabidopsis* Col-0 plants is suppressed at 28 °C after infection with an ETI-activating *P. syringae* strain (Extended Data Fig. 1b). Finally, elevated temperature downregulated the expression of SA-response genes in both dicot (rapeseed, tobacco and tomato) and monocot (rice) crop plants, after pathogen infection and/or pathogen-independent elicitation with benzothiadiazole (BTH), a synthetic SA analogue (Extended Data Fig. 1c–g). Together, these results suggest that the temperature vulnerability of the SA pathway is probably a common feature in plants and has pervasive effects on basal immunity, PTI and ETI.

## Independence from phyB and ELF3 thermosensors

**Extended Data Fig. 2:**
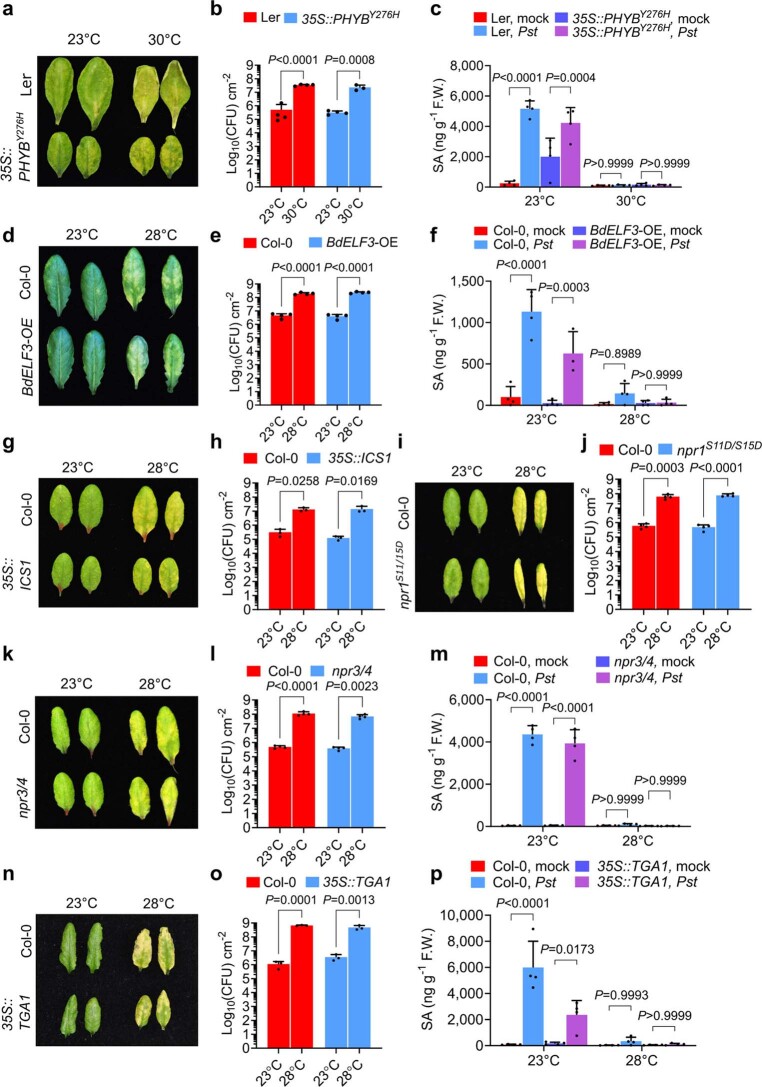
Basal resistance to *Pst* DC3000 at control (23 °C) and elevated temperature (28–30 °C) in constitutively activated phyB and ELF3 thermosensor lines and in genetically activated SA biosynthetic and signalling mutants. **a–f**, Symptom expression at 3 day post-inoculation (dpi) (**a**, **d**), *in planta Pst* DC3000 bacterial levels at 3 dpi (**b**, **e**) and SA levels of mock (0.25 mM MgCl_2_)- and *Pst* DC3000-inoculated leaves [1.0 x 10^6^ Colony Forming Units (CFU) mL^−1^] at 1 dpi (**c**, **f**) of Ler (**a**–**c**), Col-0 (**d**–**f**), *35S::PHYB*^*Y276H*^ (**a**–**c**), and *BdELF3-*OE (**d**–**f**). **g–j**, Symptom expression at 3 dpi (**g**, **i**) and *in planta Pst* DC3000 bacterial levels at 3 dpi (**h**,** j**) of Col-0 (**g**–**j**), *35S::ICS1* (**g**, **h**), and *npr1*^*S11D/S15D*^ (**i**, **j**). **k–p**, Symptom expression at 3 dpi (**k**,**n**), *in planta Pst* DC3000 bacterial levels at 3 dpi (**l**, **o**) and SA levels of mock- and *Pst* DC3000-inoculated leaves at 1 dpi (**m**, **p**) of Col-0 (**k**–**p**), *npr3/4* (**k**–**m**), and *35S::TGA1* (**n**–**p**). Results show the means ± S.D. [n = 4 (**c**, **e**, **j**, **l**, **m**, **p**) or n = 3 (**h**, **o**) biological replicates] from one representative experiment (of three independent experiments) analyzed with two-way ANOVA with Tukey’s HSD for significance. Results show the means ± S.D. [(**b**) n = 4 biological replicates except *35S::PHYB*^*Y276H*^ at 30 °C (n = 3 biological replicates), (**f**) n = 4 biological replicates except *BdELF3*-OE, *Pst* at 23 °C (n = 3 biological replicates)] from one representative experiment (of three independent experiments) analyzed with two-way ANOVA with Tukey’s HSD for significance. Exact *P*-values for all comparisons are detailed in the Source Data files Source data

Recent studies showed that phyB^8,9^ and ELF3^10^ regulate thermo-responsive plant growth and development. To determine whether heat wave suppression of SA production also occurs via these thermosensing mechanisms, we tested constitutively activated phyB (*35S::PHYB*^*Y276H*^)^8^ or ELF3 thermosensor (*BdELF3-*OE)^10^ lines that do not exhibit thermo-responsive growth. However, these plants remained temperature-sensitive in pathogen-induced SA accumulation and displayed increased bacterial susceptibility at 28 °C (Extended Data Fig. 2a–f). These results indicate that SA suppression at elevated temperature is independent of phyB or ELF3 thermosensing mechanisms. This agrees with our previous study showing that neither activated phyB nor quadruple mutants in *PHYTOCHROME-INTERACTING FACTORS* (*pif*) conferred temperature-resilient basal immunity to *Pst* DC3000 infection during a simulated heat wave^15^.

## Beyond SA biosynthesis and receptor genes

Because *ICS1* expression is crucial for SA production^21^ and is downregulated at elevated temperature^15^, we next tested whether downregulated *ICS1* (Fig. 1b) is the rate-limiting step controlling heat wave-mediated SA suppression. Surprisingly, although constitutive *ICS1* expression from the 35S cauliflower mosaic virus (CaMV) promoter resulted in constitutive SA accumulation at 23 °C, as expected, it did not restore pathogen-induced SA at 28 °C and the *ICS1*-overexpressing plants showed compromised basal immunity at 28 °C, just like wild-type Col-0 plants (Fig. 1d and Extended Data Fig. 2g,h). SA accumulation is also regulated by the SA receptors^3,26^ (NPR proteins); however, constitutive NPR1 activation using *npr1*^*S11D/S15D*^ phosphomimetic lines^27^ did not restore SA accumulation, and these plants exhibited hypersusceptibility to *Pst* DC3000 at 28 °C (Fig.1e and Extended Data Fig. 2i,j). Finally, removal of antagonistic SA receptors NPR3 and NPR4 using the *npr3 npr4* mutant^26^ also could not counter suppression of SA immunity at elevated temperature (Extended Data Fig. 2k–m).

Overall, these results highlighted the challenges to identification of the primary, rate-limiting step in the SA pathway that is affected by heat waves based on well-established plant thermosensing^8–10^ and SA biosynthesis–receptor^3,21,26–28^ paradigms.

## Effect on *CBP60g* and *SARD1* expression

**Extended Data Fig. 3:**
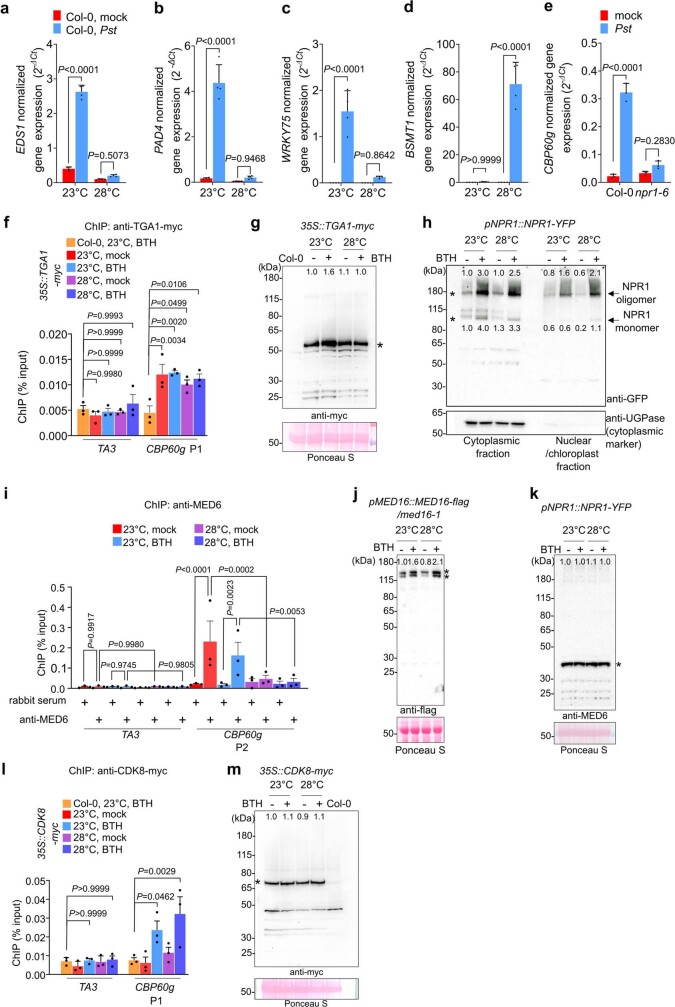
Effect of elevated temperature on transcript levels, protein levels and promoter recruitment of SA pathway regulators. **a–d**, Endogenous *EDS1* (**a**)*, PAD4* (**b**)*, WRKY75* (**c**), and *BSMT1* (**d**) transcript levels of samples in Fig. 1b at 24 h after treatment (1 dpi). **e**, *CBP60g* gene expression levels in Col-0 and *npr1*–*6* plants at 24 h after *Pst* DC3000 inoculation [1.0 x 10^6^ Colony Forming Units (CFU) mL^−1^] at 23 °C. **f**, ChIP-qPCR analysis of *35S::TGA1-4myc* using anti-myc antibody and primer sets indicated in Fig.2f. Binding of TGA1-myc to *CBP60g* locus is not affected by temperature in mock (0.1% DMSO)- or BTH-treated samples (*P*-value = 0.7903 and 0.9566, respectively). **g**, Immunoblot results of *35S::TGA1-myc* used for ChIP-qPCR analyses in (**f**). **h**, NPR1 immunoblot of *NPR1pro::NPR1-YFP* plant cytosolic and nuclear protein fractions 24 h after BTH treatment at 23 °C and 28 °C. Both NPR1 oligomers (high molecular weight) and monomers (low molecular weight) are indicated by arrowheads. Anti-UGPase immunoblot is shown as the cytoplasmic marker control. **i**, ChIP-qPCR results of *NPR1pro::NPR1-YFP* using anti-MED6 antibody and primer sets indicated in Fig.2f. **j**, Immunoblot result of *MED16pro::MED16-3flag* used for ChIP-qPCR analysis in Fig. 2e. **k**, Immunoblot results of *NPR1pro::NPR1-YFP* using anti-MED6 antibody used for ChIP-qPCR analyses in (**i**). **l**, ChIP-qPCR results of *35S::CDK8-myc* using anti-myc antibody and primer sets indicated. **m**, Immunoblot results of *35S::CDK8-myc* using anti-myc antibody used for ChIP-qPCR analyses in (l). For immunoblot (**g**, **j**, **k**, **m**), stained RuBisCO large subunits are shown as loading controls. Numbers in panels (**g**, **h**, **j**, **k**, **m**) indicate relative protein band signal intensities compared to the corresponding band denoted with a * symbol(s). For gel/blot source data, see Supplementary Fig. 1. For ChIP analyses, the *TA3* transposon was used as the negative control target locus. Primer positions (P1 for promoter region and P2 for coding region) are indicated in Fig. 2f. Antibody information is included in Supplementary Table 5. Result in (**a**–**e**) shows the means ± S.D. [n = 4 (**a**–**d**) or 3 (**e**) biological replicates] from one representative experiment [of three (**a**–**d**) or two (**e**) independent experiments] analyzed with two-way ANOVA with Tukey’s HSD for significance. Results in (**g**, **h**, **j**, **k**, **m**) show one representative experiment [of two (**g**, **h**) or three (**j**, **k**, **m**) independent experiments]. Results in (**f**, **i**,** l**) are the average ± S.D. [of three independent experiments (n = 3 experiments)], analyzed with two-way ANOVA with Tukey’s HSD for significance. Exact *P*-values for those comparisons that are greater than 0.05 are detailed in the Source Data files Source data

The inability of constitutive *ICS1* expression and NPR1 receptor activation to restore SA production at elevated temperature (Fig. 1d,e) led us to pursue a different strategy. We performed RNA sequencing of *Pst* DC3000-infected Col-0 plants at normal and elevated temperatures. In addition to *ICS1*, pathogen induction of various SA-associated defence regulators was suppressed at 28 °C (Supplementary Table 1, cluster A and Supplementary Data 2), including *EDS1*, *PAD4* and *WRKY75* (Extended Data Fig. 3a–c), whereas the SA catabolic gene *BSMT1* was upregulated at 28 °C (Extended Data Fig. 3d). Genes that were downregulated by elevated temperature in cluster A included *CBP60g* (Fig. 1f) and *SARD1* (Supplementary Data 2), which encode functionally redundant *ICS1* promoter-binding transcription factors required for SA production^29–31^. Monitoring a *GUS* reporter fused to the *CBP60g* promoter also detected decreased transcript levels at 28 °C (Fig. 1g), indicating that elevated temperature affects *CBP60g* expression mainly through transcription. Further examination revealed that numerous CBP60g and SARD1 target genes^31^ were suppressed at 28 °C (Fig. 1h), including many known crucial regulators of basal and systemic immunity (Fig. 1i), raising the possibility that expression of *CBP60g* or *SARD1* may be the primary target in SA suppression at elevated temperature.

## Thermosensitive GDACs and GBPL3 binding

**Fig. 2:**
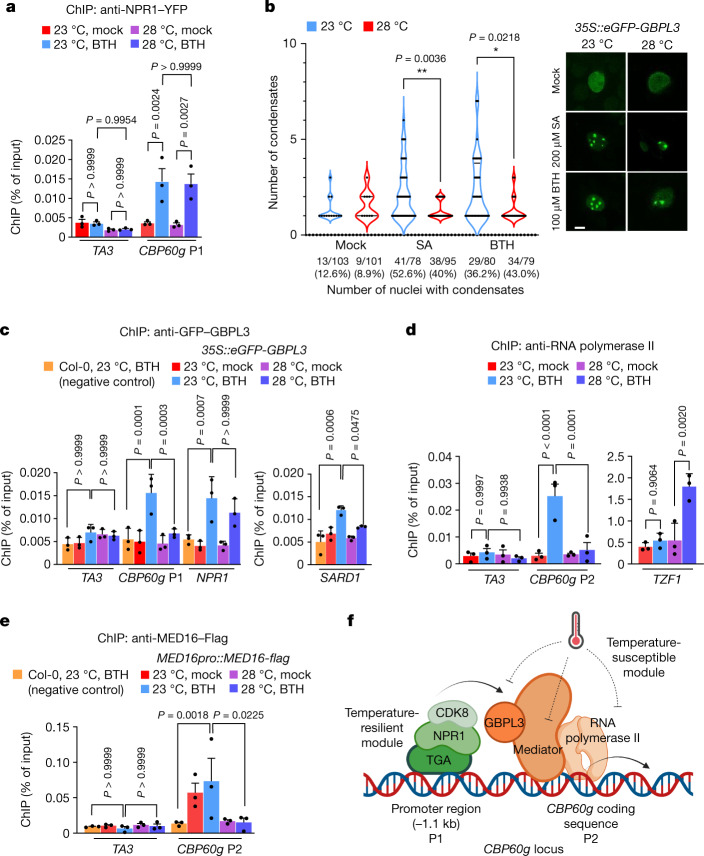
Elevated temperature represses *CBP60g* promoter activity. Four- to five-week-old Col-0 and indicated transgenic plants were treated with mock (0.1% DMSO) or 100 µM BTH solution and then incubated at 23 °C and 28 °C. ChIP–qPCR and confocal imaging were performed in plants one day after treatment. **a**, ChIP–qPCR analyses of *NPR1pro::NPR1-YFP* using anti-GFP antibody and indicated primer sets. The position of the *CBP60g* primer sequence is shown in **f**. **b**, Confocal imaging of eGFP–GBPL3 in *35S::eGFP-GBPL3* infiltrated with mock (0.1% DMSO), 200 µM SA or 100 µM BTH solution at 23 °C or 28 °C 1 day after treatment. Scale bar, 10 μm. **c**–**e**, ChIP–qPCR analyses of *35S::eGFP-GBPL3* (**c**), *NPR1pro::NPR1-YFP* (**d**) and *MED16pro::MED16-flag* (**e**) plants using the indicated antibodies and primer sets. **f**, Schematic showing known regulators binding at the *CBP60g* locus. Temperature-susceptible (green) and temperature-resilient (orange) modules are indicated. Primer positions (P1 for promoter region and P2 for coding region) are indicated. For ChIP analyses, the *TA3* transposon was used as the negative control target locus in (**a**,**c**–**e**). A BTH-treated Col-0 sample incubated at 23 °C (**c**,**e**) was used as a negative control for immunoprecipitation. Results in (**a**,**c**–**e**) are mean ± s.d. of three independent experiments; two-way ANOVA with Tukey’s HSD. Images in **b** show one representative experiment (of four independent experiments); one-way ANOVA with Bartlett’s test. Exact *P*-values greater than 0.05 are shown in the Source Data. Source data

To understand the mechanism by which elevated temperature affects *CBP60g* transcription, we investigated the effect of elevated temperature on known regulators of *CBP60g*. The current SA signalling model suggests that NPR receptors interact with TGACG-binding (TGA) transcription factors^3,26,28^, which regulate *CBP60g* gene expression (Extended Data Fig. 3e) and SA biosynthesis. However, we found that constitutive *TGA1* expression did not restore SA levels at elevated temperature and that *35S::TGA1* plants still exhibited temperature-sensitive basal immunity to *Pst* DC3000 (Extended Data Fig. 2n–p). In agreement, TGA1 binding to the *CBP60g* promoter and total TGA1 protein levels were not affected at 28 °C (Extended Data Fig. 3f,g). Similarly, NPR1 recruitment to the *CBP60g* promoter was similar at 23 °C and 28 °C after chromatin immunoprecipitation (ChIP) (Fig. 2a). Consistent with this result, NPR1 monomerization, which is associated with NPR1 function^32^, was similar at both temperatures (Extended Data Fig. 3h). Together, these results pointed to an NPR1- and TGA1-independent mechanism for suppressing *CBP60g* transcription and SA production at elevated temperature.

**Extended Data Fig. 4:**
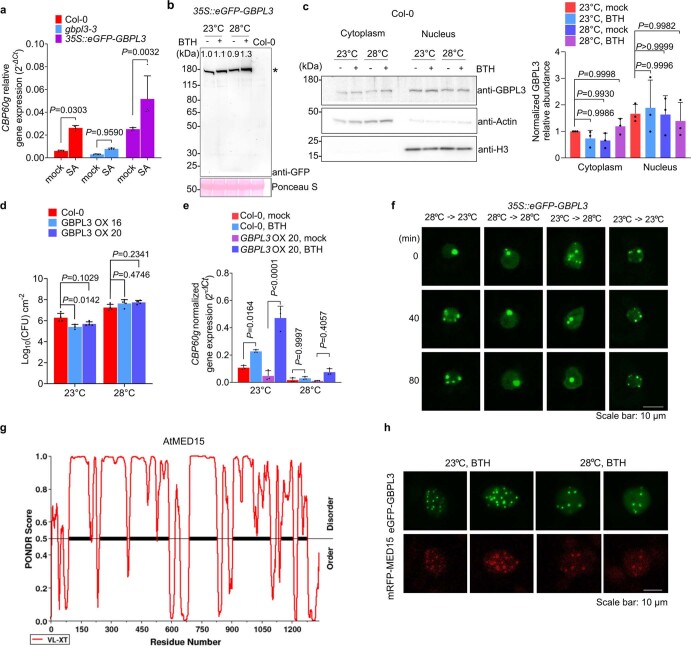
Characterization of *35S::eGFP-GBPL3* and *GBPL3 OX* plants. **a**, *CBP60g* gene expression levels in Col-0, *gbpl3-3*, and *35S::eGFP-GBPL3* plants at 24 h (1 day) after mock (water) or 200 µM salicylic acid spray at 23 °C. **b**, Immunoblot results of *35S::eGFP-GBPL3* used for ChIP-qPCR analyses in Fig. 2c. Stained RuBisCO large subunits are shown as loading controls. Numbers in the panel indicate relative protein band signal intensities compared to the corresponding band denoted with a * symbol. **c**, Subcellular fractionation of *Arabidopsis* Col-0 leaf cells treated with mock (0.1% DMSO) or BTH for 24h at control (23 °C) or elevated temperature (28 °C). Actin and Histone H3 protein were used as markers of cytoplasmic and nuclear fractions, respectively. **d**, *In planta Pst* DC3000 [1.0 x 10^6^ Colony Forming Units (CFU) mL^−1^] bacterial levels in Col-0, *GBPL3* OX #16 and *GBPL3* OX #20 plants at 3 dpi. **e**, *CBP60g* gene expression levels of Col-0 and *GBPL3* OX #20 plants at 24 h after mock (0.1% DMSO) or 100 µM BTH spray at 23 °C or 28 °C. **f**, Time lapse confocal microscopy of *Arabidopsis* mesophyll cell expressing eGFP-GBPL3 after transfer to 28 °C from 23 °C or to 23 °C from 28 °C. Scale bar, 10 µm. **g**, Prediction of intrinsically disordered region in AtMED15 (Threshold score: 0.5). **h**, Confocal microscopy of *Nicotiana tabacum* mesophyll cells transiently expressing eGFP-GBPL3 and mRFP-MED15 at 23 °C and 28 °C. Six to seven weeks old *N. tabacum* leaves were infiltrated with *Agrobacterium* harbouring *35S::eGFP-GBPL3* or *35S::mRFP-MED15-flag*. After incubation for 3 days at control temperature, the plants were treated with 100 µM BTH solution and shifted to 23 °C or 28 °C. After 1 day, mesophyll cells were visualized by confocal microscopy. Scale bar, 10 µm. Results in (**a**, **d**, **e**) show the means ± S.D. [(**a**) n = 4, (**d**) n = 4 except GBPL3 OX 16 at 23 °C (n = 3 biological replicates), or (**e**) n = 3 biological replicates] from one representative experiment (of two independent experiments), analyzed with two-way ANOVA with Tukey’s HSD for significance. Results in (**b**, left panel of **c**) show one representative experiment (of three independent experiments). Result in (right panel of **c**) shows the means ± S.D. (of three independent experiments) analyzed with one-way ANOVA with Bartlett’s test for significance. Results in (**f**, **h**) show one representative experiment (of two independent experiments). Exact *P*-values for all comparisons are detailed in the Source Data files Source data

GBPL3 is a key positive regulator of SA signalling and immunity^11^. We found that GBPL3 is required for *CBP60g* gene expression in response to SA (Extended Data Fig. 4a). GBPL3 has been proposed to act on promoters via phase-separated biomolecular condensates together with Mediator and RNA polymerase II^11^ (Pol II). The thermosensor ELF3 contains an intrinsically disordered domain (IDR) that is involved in condensate formation and temperature sensing^10^. GBPL3 also contains an IDR, which mediates intranuclear GDAC formation^11^. We therefore tested whether elevated temperature negatively affects GDAC formation and/or GBPL3 recruitment to the *CBP60g* promoter, which is required for *CBP60g* transcription. Indeed, the number of GDACs per nucleus was significantly reduced at 28 °C compared with 23 °C (Fig. 2b). Experiments using ChIP with quantitative PCR (ChIP–qPCR) revealed that GBPL3 binding to the promoters of *CBP60g* and its functionally redundant paralogue *SARD1* were markedly reduced at 28 °C in BTH-treated plants (Fig. 2c), even though total GBPL3 protein levels remained similar at both temperatures (Extended Data Fig. 4b,c). Consistent with the observation that the temperature effect is not at the level of GBPL3 expression, *GBPL3* overexpression did not restore *CBP60g* expression (Extended Data Fig. 4d,e). Notably, time-lapse imaging revealed that GDACs appeared reversibly at 23 °C or disappeared at 28 °C in response to temperature shifts, indicating that their formation and dissolution are temporally dynamic (Extended Data Fig. 4f). Furthermore, MED15—another component of the GDAC^11^ that contains multiple IDRs (Extended Data Fig. 4g)—also showed temperature sensitivity (Extended Data Fig. 4h). GBPL3 and MED15 were co-localized in individual GDACs, as observed previously^11^, and they either appeared or disappeared together in response to elevated temperature.

## GPBL3 specificity on *CBP60g* and *SARD1* loci

We found that elevated temperature-mediated suppression of GBPL3 recruitment occurs selectively at certain loci, but not at all GBPL3 target sites. For example, elevated temperature suppressed GBPL3 recruitment to *CBP60g* and *SARD1*, but not to *NPR1* (Fig. 2c), which is consistent with temperature-resilient *NPR1* transcript levels^15^. Of note, we observed that despite a significantly reduced number of GDACs per nucleus, elevated temperature did not decrease the number of nuclei that contained GDACs (Fig. 2b). Collectively, our data indicate that there appear to be two subpopulations of GDACs in vivo. One subpopulation is sensitive to 28 °C (the one associated with GBPL3 recruitment to the *CBP60g* promoter) and the other is insensitive to 28 °C (the one associated with GBPL3 recruitment to the *NPR1* promoter).

Next, we investigated whether altered GBPL3 condensate formation and reduced GBPL3 binding to the *CBP60g* promoter at 28 °C is linked to impaired recruitment of Pol II and Mediator subunits. As shown in Fig. 2d, elevated temperature suppressed BTH-induced Pol II association with the *CBP60g* promoter, but not with the promoter of a control gene *TZF1*, which is highly induced by BTH at elevated temperature^15^. Furthermore, elevated temperature significantly reduced *CBP60g* promoter binding by MED16, a Mediator tail subunit associated with SA gene expression^33^ (Fig. 2e). Binding of the Mediator head subunit MED6 to the *CBP60g* promoter was also significantly reduced at 28 °C compared with 23 °C (Extended Data Fig. 3i). Differential Mediator subunit recruitment was not owing to changes in protein abundance, since protein levels of MED16 and MED6 remained the same at 23 °C and 28 °C (Extended Data Fig. 3j,k). Notably, not all Mediator components were affected at elevated temperature, as the level and binding of CDK8—a Mediator kinase module subunit that interacts with NPR1 to regulate SA signalling^34^—were similar at 23 °C and 28 °C (Extended Data Fig. 3l,m). These results indicate that elevated temperature selectively affects the recruitment of GBPL3 and several SA pathway-relevant Mediator complex subunits to the *CBP60g* promoter, independently of the NPR1–TGA1–CDK8 module (Fig. 2f).

## *CBP60g* and *SARD1* expression is rate-limiting

**Fig. 3:**
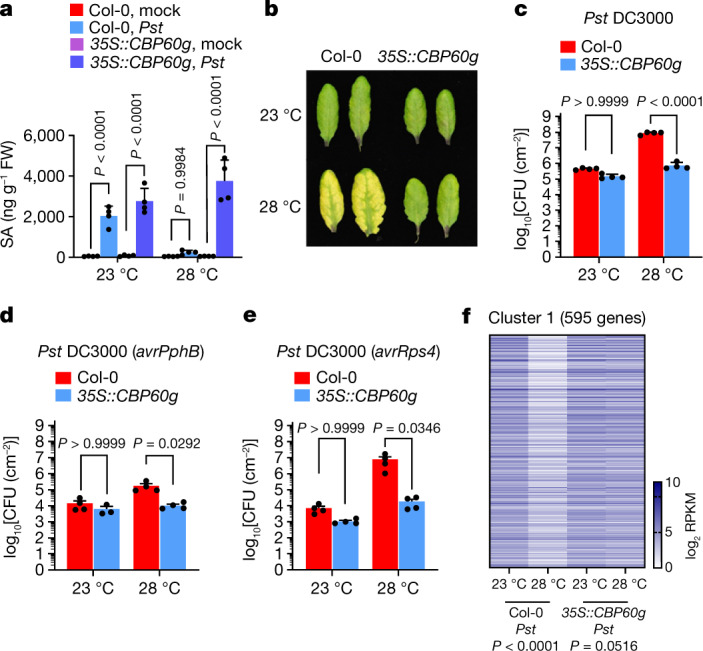
Restoration of SA accumulation and immunity at elevated temperature in *35S::CBP60g* plants. Wild-type Col-0 and *35S::CBP60g* plants were syringe-infiltrated with mock (0.25 mM MgCl_2_) or *Pst* DC3000 (10^6^ CFU ml^−1^) and incubated at 23 °C or 28 °C. **a**, SA levels in mock- and *Pst* DC3000-inoculated plants at 24 h (1 dpi). **b**, Images of leaves from *Pst* DC3000-inoculated plants at 3 dpi. **c**, In planta *Pst* DC3000 bacterial levels at 3 dpi. **d**,**e**, In planta *Pst* DC3000 (*avrPphB*) (**d**) or *Pst* DC3000 (*avrRps4*) (**e**) ﻿bacterial levels at 3 dpi. **f**, Heat map of RNA-seq reads for genes that are downregulated in Col-0 grown at 28 °C but fully or partially restored in *35S::CBP60g* grown under the same conditions. RPKM, reads per kilobase of transcript per million mapped reads. Data in **a**,**c**,**e** are mean ± s.d. (*n* = 4 biological replicates) of one representative experiment (out of three independent experiments) analysed by two-way ANOVA with Tukey’s HSD. Results in **d** are mean ± s.d. (*n* = 4 biological replicates except *35S::CBP60g* at 23 °C (*n* = 3 biological replicates)) of one representative experiment (out of three independent experiments) analysed by two-way ANOVA with Tukey’s HSD. Exact *P*-values for all comparisons are shown in the Source Data. Source data

**Extended Data Fig. 5:**
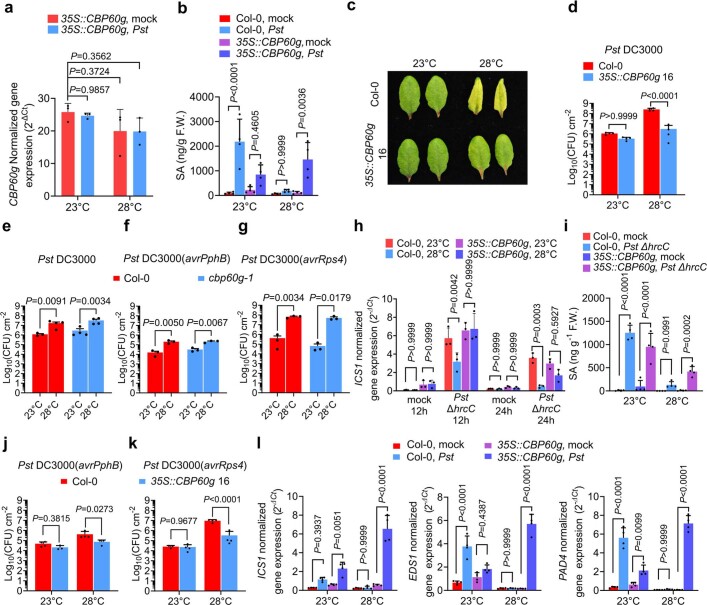
Characterization of *35S::CBP60g* 16 and *cbp60g-1* plants. **a**, *CBP60g* transcript levels in 4-week old *35S::CBP60g* at 23 °C or 28 °C 1 day after mock (0.25 mM MgCl_2_) treatment or *Pst* DC3000 infection [1.0 x 10^6^ Colony Forming Units (CFU) mL^−1^]. **b–d**, SA levels at 1 dpi (**b**), symptom expression at 3 dpi (**c**) and *in planta Pst* DC3000 [1.0 x 10^6^ Colony Forming Units (CFU) mL^−1^] bacterial levels at 3 dpi (**d**) of Col-0 and *35S::CBP60g* 16 plants. **e–g**, bacterial levels in Col-0 and *cbp60g-1* plants inoculated with *Pst* DC3000 (**e**), *Pst* DC3000 (*avrPphB*) (**f**), and *Pst* DC3000 (*avrRps4*) (**g**) at 3 dpi. **h**, *ICS1* gene expression levels in *Pst* DC3000 *ΔhrcC-*infected Col-0 and *35S:CBP60g* plants [1.0 x 10^8^ Colony Forming Units (CFU) mL^−1^] at 12- and 24-h post-inoculation (hpi). **i**, SA levels in *Pst* DC3000 *ΔhrcC-*infected Col-0 and *35S:CBP60g* plants (1.0 x 10^8^ Colony Forming Units (CFU) mL^−1^) at 24 h post-inoculation. **j–k**, *In planta Pst* DC3000 (*avrPphB*) (**j**), and (*avrRps4*) (**k**) bacterial levels of Col-0 and *35S::CBP60g* 16 plants at 3 dpi. **l**, *ICS1, EDS1* and *PAD4* gene expression levels of Col-0 and *35S::CBP60g* plants 1 day after mock (0.25 mM MgCl_2_)- and *Pst* DC3000-infiltration [1.0 x 10^6^ Colony Forming Units (CFU) mL^−1^]. Results show the means ± S.D. [n = 3 (**a**, **f**, **g**, **h**) or 4 (**b**, **d**, **i**) biological replicates] from one representative experiment [of two (**a**, **h**, **i**) or three (**b**, **d**, **f**, **g**) independent experiments] analyzed with two-way ANOVA with Tukey’s HSD for significance. Results in (**e**) show the means ± S.D. [n = 4 biological replicates except Col-0 at 23 °C (n = 3 biological replicates)] from one representative experiments (of three independent experiments) analyzed with two-way ANOVA with Tukey’s HSD for significance. Results in (**j**) show the means ± S.D. [n = 4 (Col-0) or 3 (*35S::CBP60g* 16) biological replicates] from one representative experiments (of three independent experiments) analyzed with two-way ANOVA with Tukey’s HSD for significance. Results in (**k**) show the means ± S.D. [n = 4 biological replicates except *35S::CBP60g* 16 at 23 °C (n = 3 biological replicates)] from one representative experiments (of three independent experiments) analyzed with two-way ANOVA with Tukey’s HSD for significance. Exact *P*-values for those comparisons that are greater than 0.05 are detailed in the Source Data files Source data

**Extended Data Fig. 6:**
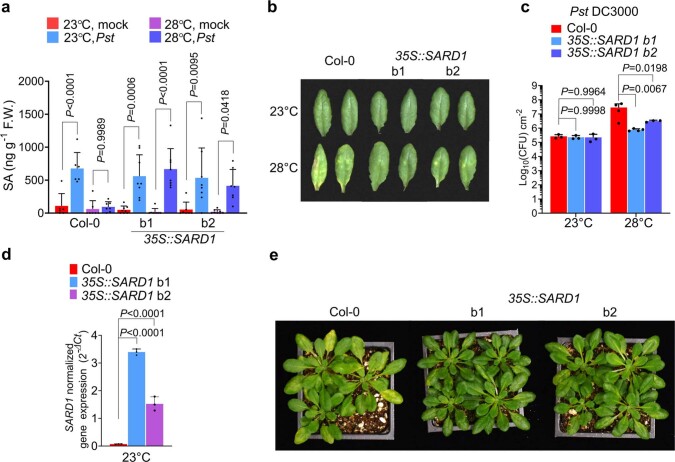
Characterization of *35S::SARD1* plants. **a–c**, SA levels at 24 h (**a**), symptom expression at day 3 (**b**), *in planta* bacterial levels at day 3 (**c**) post-inoculation with mock (0.25 mM MgCl_2_) or *Pst* DC3000 solution [1.0 x 10^6^ Colony Forming Units (CFU) mL^−1^]. **d**, *SARD1* gene expression levels in 4-week-old plants of Col-0 and *35S::SARD1*. **e**, Appearance of 4.5-week-old Col-0 and *35S::SARD1* plants (lines b1 and b2) grown at 23 °C were infiltrated with 1 x 10^6^ CFU mL^−1^ *Pst* DC3000 and further incubated at 28 °C for 3 days. Results in (**a**) show the means ± S.D. [n = 6 (Col-0, 23 °C mock and Col-0, 23 °C *Pst*), 7 (Col-0, 28 °C mock and Col-0, 28 °C *Pst*), 8 (all *35S::SARD1* b1 line data), 7 (*35S::SARD1* b2 line, 23 °C mock), 8 (*35S::SARD1* b2 line, 23 °C *Pst*), 8 (*35S::SARD1* b2 line, 28 °C mock), or 7 (*35S::SARD1* b2 line, 28 °C *Pst*) biological replicates from two independent experiments] analyzed with two-way ANOVA with Tukey’s HSD for significance. Results in (**c**) show the means ± S.D. [n = 3 (Col-0 at 23 °C), 4 (Col-0 at 28 °C), 3 (*35S::SARD1* b1 at 23 °C), 4 (*35S::SARD1* b1 at 28 °C), or 3 (*35S::SARD1* b2 at 23 °C and 28 °C) biological replicates] from one representative experiments (of four independent experiments) analyzed with two-way ANOVA with Tukey’s HSD for significance. Results in (**d**) show the means ± S.D. (n = 3 biological replicates) from one representative experiments (of two independent experiments) analyzed with one-way ANOVA with Bartlett’s test for significance. Result in (**e**) shows one representative experiment of four independent experiments. Exact *P*-values for those comparisons that are greater than 0.05 are detailed in the Source Data files Source data

The identification of *CBP60g* and *SARD1* transcription as the primary thermo-sensitive step in the SA pathway downstream of GBPL3 prompted us to test whether expression of *CBP60g* and *SARD1* is a rate-limiting step for SA production at elevated temperature and, if so, whether restoring *CBP60g* and *SARD1* expression would sufficiently render SA production resilient to increased temperature. Unlike expression of the activated SA receptor gene *NPR1* or the SA biosynthetic gene *ICS1* (Fig. 1d,e and Extended Data Fig. 2g–j), *35S::CBP60g* and *35S::SARD1* lines restored pathogen-induced SA production and maintained basal immunity to *Pst* DC3000 at 28 °C, in contrast to Col-0 plants (Fig. 3a–c and Extended Data Figs. 5a–d and 6a–e). Because CBP60g and SARD1 are functionally redundant^35^, temperature-sensitive immunity to *Pst* DC3000 remained in the *cbp60g* single mutant, as expected (Extended Data Fig. 5e–g).

**Extended Data Fig. 7:**
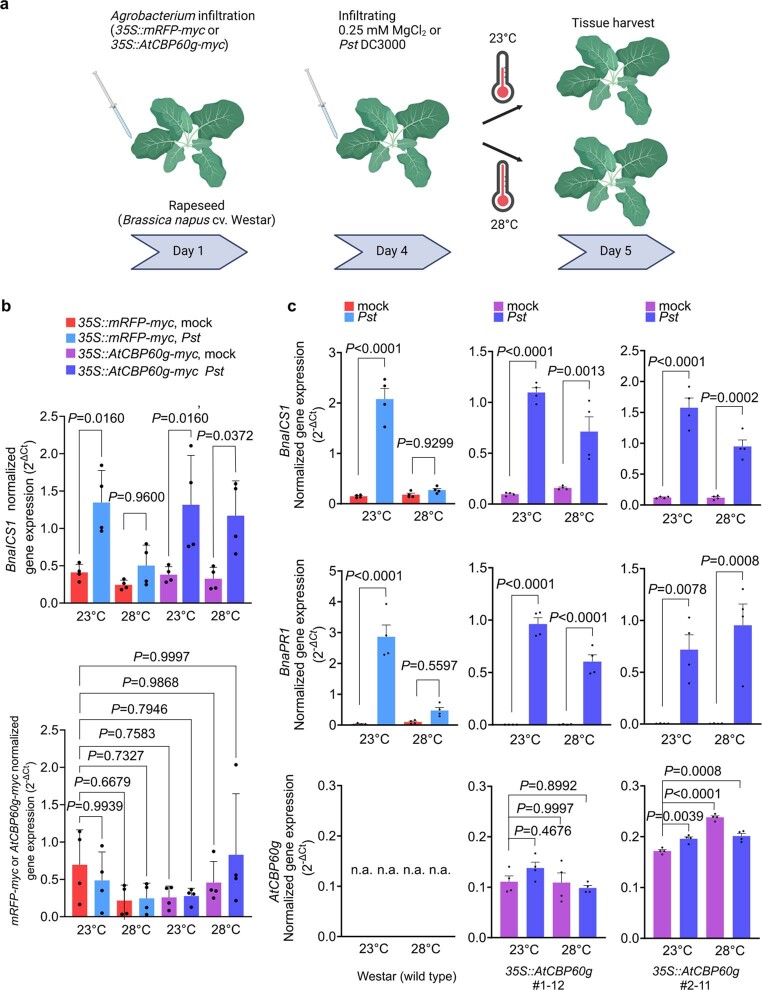
*BnaICS* and *BnaPR1* transcript levels in transgenic rapeseed plants expressing *AtCBP60g-myc*. **a**, A schematic diagram of experimental flow using *Agrobacterium*-mediated transient expression system. **b**, Transcript levels of *BnaICS1* and myc-tagged transgenes (*mRFP-myc* or *AtCBP60g-myc*) in mock (0.25 mM MgCl_2_)- or *Pst* DC3000-infiltrated [1.0 x 10^5^ Colony Forming Units (CFU) mL^−1^] rapeseed leaves at 1 dpi. Leaves were pre-infiltrated with *Agrobacterium* suspension 3 days before mock or *Pst* DC3000 treatment. Results in (**b**) are the means ± S.D. (n = 4 biological replicates from two independent experiments). Statistical analysis was performed using two-way ANOVA with Tukey’s HSD. The experiment was repeated four times with similar results. **c**, Transcript levels of *BnaICS1*, *BnaPR1* and *AtCBP60g-myc* in mock- or *Pst* DC3000-infiltrated [1.0 x 10^5^ Colony Forming Units (CFU) mL^−1^] wild-type and two independent *35S::AtCBP60g-myc* transgenic rapeseed leaves. *AtCBP60g* transcript level in each leaf sample was quantified (bottom row). No *AtCBP60g* transcript was detected in Westar samples as control, whereas *AtCBP60g* transcript was detected in each *35S::AtCBP60g-myc* sample. Data in (**c**) are the means S.E.M. (n = 4 biological replicates). The experiment was repeated twice. Statistical analysis was performed using two-way ANOVA with Tukey’s HSD. n.a., not applicable. Exact *P*-values for those comparisons that are greater than 0.05 are detailed in the Source Data files Source data

In addition to restoring basal immunity to the virulent pathogen *Pst* DC3000, the temperature-resilient SA production and gene expression in *35S::CBP60g* plants extends to infection by the non-pathogenic strain *Pst ΔhrcC*, which activates PTI in vivo (Extended Data Fig. 5h,i), and to infection by ETI-activating *Pst* DC3000(*avrPphB*) and *Pst* DC3000(*avrRps4*)^28,36^ (Fig. 3d,e and Extended Data Fig. 5j,k). Because ETI is widely used to guard crops against pathogens and insects^28,36^, these results suggest potentially broad applications of restoring *CBP60g* expression to counter suppression of not only basal immunity to virulent pathogens, but also ETI at elevated temperature. Finally, as shown in Extended Data Figs. 1 and 7, elevated temperature downregulated SA-response gene expression in both *Arabidopsis* and in crop plants such as tobacco and rapeseed. Both transient and stable *AtCBP60g* expression substantially restored *Pst* DC3000-induced expression of the *ICS1* and *PR1* orthologues *BnaICS1* and *BnaPR1*, respectively, in rapeseed leaves at elevated temperature (Extended Data Fig. 7a–c).

**Extended Data Fig. 8:**
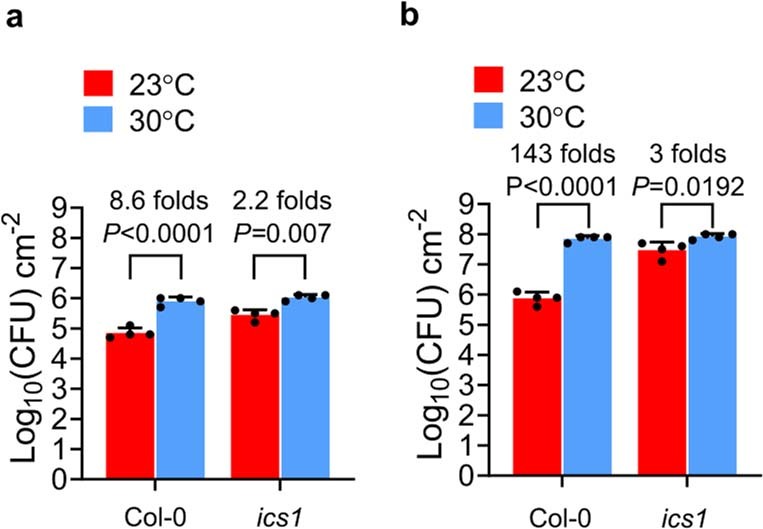
*Pst* DC3000 bacterial population levels in *Arabidopsis* Col-0 and the *ics1* mutant. **a–b**, *In planta Pst* [1.0 x 10^6^ Colony Forming Units (CFU) mL^−1^] bacterial levels in Col-0 and *ics1* (i.e., *sid2-2*) plants at 23 °C and 30 °C at 1 (**a**) and 3 (**b**) dpi. Data are the means ± S.D. (n = 4 biological replicates). The experiment was repeated three times. Statistical analysis was performed using two-way ANOVA with Tukey’s HSD. Exact *P*-values for all comparisons are detailed in the Source Data files Source data

Consistent with their immune phenotypes, *35S::CBP60g Arabidopsis* plants had restored pathogen-induced expression of CBP60g target genes *ICS1*, *EDS1* and *PAD4* at 28 °C (Extended Data Fig. 5l). Further RNA sequencing of pathogen-inoculated Col-0 and *35S::CBP60g* plants at 23 °C and 28 °C identified additional downregulated immunity genes that were also substantially restored in *35S::CBP60g* plants (Fig. 3f and Supplementary Data 3 and 4). This included SA biosynthesis genes *ICS1* and *PBS3* as well as the pattern recognition receptor genes *RLP23* and *LYK5*, the PTI signalling gene *MKK4*, and the pipecolic acid biosynthesis gene *ALD1* (Fig. 1i and Supplementary Data 2). Thus, *35S::CBP60g* seems to safeguard other defence modules besides SA biosynthesis, consistent with previous observations that CBP60g is a master transcription factor regulating diverse sectors of the plant immune system^31^. In line with this notion, SA-deficient *ics1* mutant plants (*sid2-2*) still exhibit some temperature sensitivity, albeit much less than wild-type Col-0 plants^15^ (Extended Data Fig. 8a,b). This more general role of CBP60g in the plant immune system may partly explain why *35S::CBP60g* plants (Fig. 3 and Extended Data Fig. 5a–d), but not *35S::ICS1* plants (Extended Data Fig. 2g,h), can recover basal immunity at 28 °C.

**Extended Data Fig. 9:**
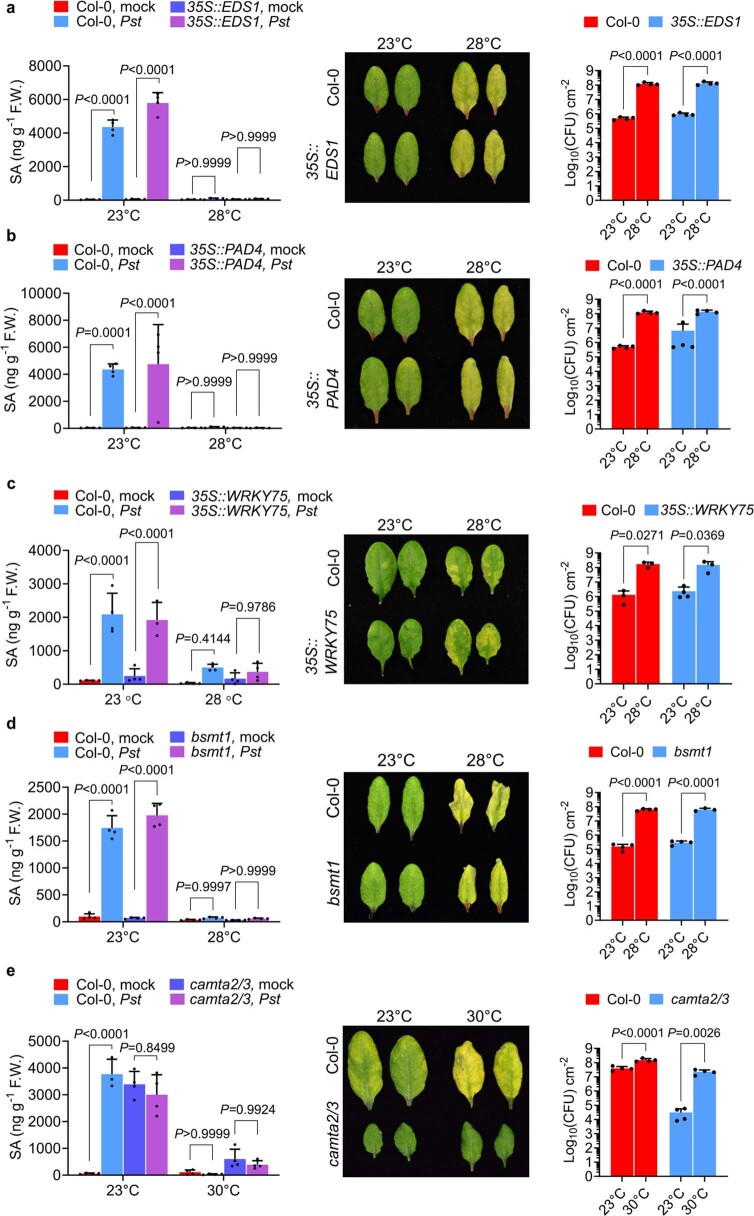
SA accumulation and basal immunity to *Pst* DC3000 at elevated temperature in plants altered in positive and negative SA regulators. **a–e**, SA levels at 1 dpi (left panels), symptom expression at 3 dpi (middle panels) and *in planta Pst* DC3000 bacterial levels at 3 dpi (right panels) of Col-0 (**a**–**e**) and *35S::EDS1* (**a**)*, 35S::PAD4* (**b**), *35S::WRKY75* (**c**), *bsmt1* (**d**) and *camta2/3* plants (**e**) [1.0 x 10^6^ Colony Forming Units (CFU) mL^−1^]. Results show the means ± S.D. [n = 4 (**a**, **b**) biological replicates] from one representative experiment (of three independent experiments) analyzed with two-way ANOVA with Tukey’s HSD for significance. Results in (**c**) show the means ± S.D. [left panel: n = 4 biological replicates except *35S::WRKY75, Pst* at 23 °C (n = 3 biological replicates); right panel: n = 3 biological replicates except *35S::WRKY75* at 23 °C (n = 4 biological replicates)] from one representative experiment (of three independent experiments) analyzed with two-way ANOVA with Tukey’s HSD for significance. Results in (**d**) show the means ± S.D. [left panel: n = 4 biological replicates; right panel: n = 4 biological replicates except *bsmt1*, *Pst* at 28 °C (n = 3 biological replicates)] from one representative experiment (of three independent experiments) analyzed with two-way ANOVA with Tukey’s HSD for significance. Results in (**e**) show the means ± S.D. [left panel: n = 4 biological replicates except Col-0, *Pst* at 23 °C (n = 3 biological replicates); right panel: n = 4 biological replicates] from one representative experiment (of three independent experiments) analyzed with two-way ANOVA with Tukey’s HSD for significance. Exact *P*-values for all comparisons are detailed in the Source Data files Source data

Notably, restoration of SA production and immunity in *35S::CBP60g*/*SARD1* plants appears to be unique among known SA pathway regulators. Constitutively expressing other elevated temperature-downregulated positive SA regulators, including *ICS1, TGA1, EDS1*, *PAD4* or *WRKY75*^3,28^, did not restore SA production or basal immunity (Extended Data Fig. 2g, h and Extended Data Figs. 2n–p and 9a–c). Similarly, loss-of-function mutations in heat-upregulated SA catabolic gene *BSMT1* and SA transcriptional repressor genes *CAMTA2/3* did not restore SA levels and basal immunity at 28 °C (Extended Data Fig. 9d,e). Additionally, we previously showed that gene mutations in jasmonate, abscisic acid or ethylene hormone pathway or DELLA-regulated PIFs, which are genetically antagonistic to the SA pathway, did not revert SA suppression by elevated temperature^15^. These results illustrate that CBP60g and SARD1 are distinct regulators of the SA pathway, and the levels of these proteins become rate-limiting for controlling ICS1-dependent and -independent immunity at elevated temperature.

## Optimization of growth versus defence

**Fig. 4:**
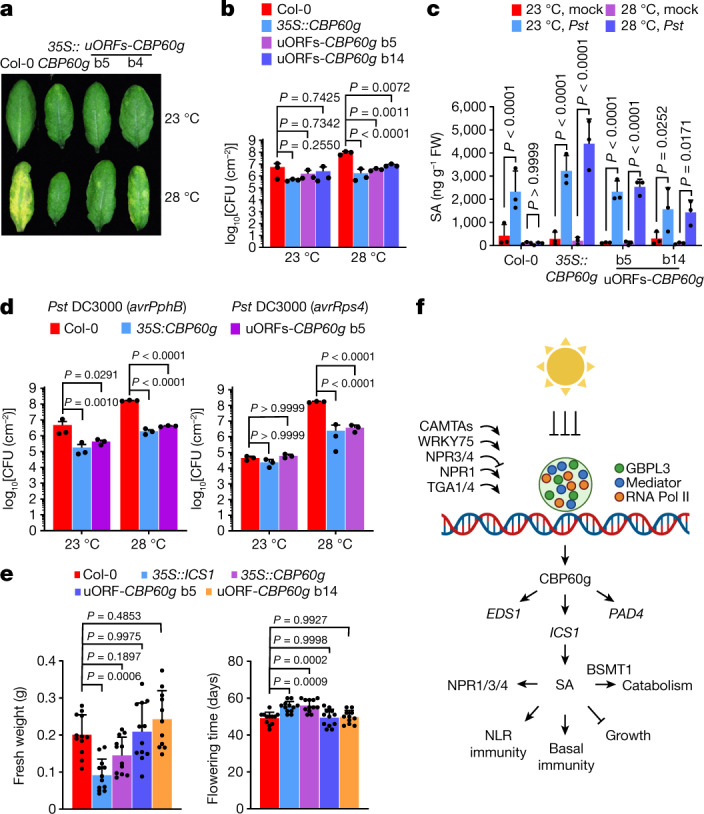
Optimized *CBP60g* expression leads to temperature-resilient SA defences without growth or developmental trade-offs. Col-0, *35S::CBP60g* and *35S::uORFs*_*TBF1*_*-CBP60g* plants were syringe-infiltrated with mock (0.25 mM MgCl_2_) or *Pst* DC3000 solution (10^6^ CFU ml^−1^) and then incubated at 23 °C and 28 °C. **a**, Foliar disease symptoms were evaluated at 3 dpi. **b**, In planta *Pst* DC3000 ﻿bacterial levels in samples in **a** at 3 dpi. **c**, SA levels in samples in **a** at 1 dpi. **d**, In planta *Pst* DC3000 (*avrPphB*) and *Pst* DC3000 (*avrRps4*) ﻿bacterial levels at 3 dpi. **e**, Fresh weight (left) at day 28 and flowering time (right) for the indicated plant genotypes. **f**, A working model of how elevated temperature targets the SA defence and immune network through *CBP60g* expression. At normal growth temperature, infection induces *CBP60g* gene expression. CBP60g regulates various defence genes, including those involved in SA accumulation (such as *ICS1, EDS1* and *PAD4*). At elevated temperature, recruitment of Mediator, GBPL3 and RNA Pol II to the *CBP60g* locus is impaired, leading to lower SA production and reduced immunity at elevated temperature. Data in **b**–**d** are mean ± s.d. (*n* =  3 biological replicates) from one representative experiment (out of three independent experiments) analysed by two-way ANOVA with Tukey’s HSD. Data in **e** are mean ± s.d. (*n* = 12 biological replicates) from one representative experiment (out of three independent experiments), analysed by one-way ANOVA with Bartlett’s test. Exact *P*-values greater than 0.05 are shown in the Source Data. Source data

**Extended Data Fig. 10:**
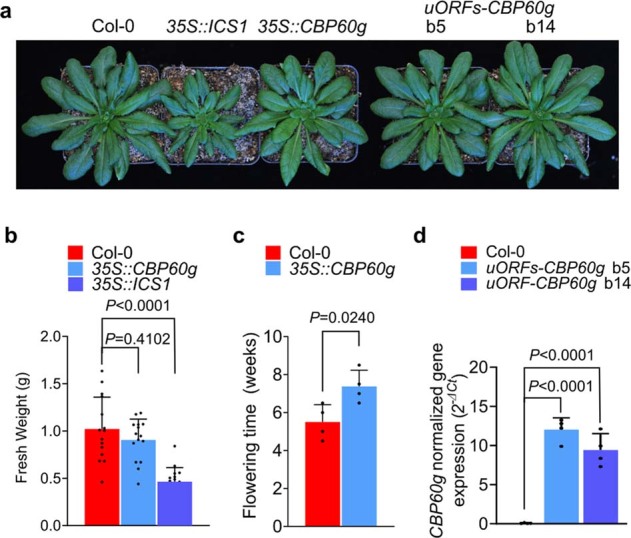
Characterization of *35S::ICS1*, *35S::CBP60g* and *uORF-CBP60g* plants. **a**, Appearance of 6-week-old Col-0, *35S::ICS1*, *35S::CBP60g* and *uORFs-CBP60g* plants. **b**, Quantification of fresh weights of 6-week-old Col-0, *35S::ICS1*, *35S::CBP60g*. **c**, Flowering time phenotypes of Col-0 and *35S::CBP60g* plants. **d**, *CBP60g* transcript levels in 4-week old Col-0, and *35S::uORFs-CBP60g* plants measured by RT-qPCR. Results in (**b**) show the means ± S.D. [n = 15 (Col-0, *35S::CBP60g*), n = 16 (*35S::ICS1*) biological replicates] from one representative experiment (of two independent experiments) analyzed with one-way ANOVA with Bartlett’s test for significance. Results in (**c**) show the means ± S.D. (n = 4 biological replicates) from one representative experiment (of two independent experiments) with two-tailed Student’s t-test. Results in (**d**) show the means ± S.D. (n = 4 biological replicates of two independent experiments) analyzed with one-way ANOVA with Bartlett’s test for significance. Exact *P*-values for all comparisons are detailed in the Source Data files Source data

A common issue with increasing expression levels of SA regulators is the inhibition of plant growth and reproduction due to the growth–defence trade-off^37,38^. This is illustrated with *35S::ICS1* plants, which have highly elevated basal SA levels at ambient temperature (Fig.1d) and show reduced growth (Extended Data Fig. 10a,b). Of note, the growth of *35S::CBP60g* and *35S::SARD1* plants was less adversely affected compared with *35S::ICS1* plants (Extended Data Figs. 6e and 10a, b), consistent with low basal SA levels in *35S::CBP60g* and *35S::SARD1* plants (Fig. 3a and Extended Data Figs. 5b and  6a). Nevertheless, detailed characterization of *35S::CBP60g* plants showed a delay in flowering (Extended Data Fig. 10c). To minimize this developmental trade-off, we expressed *CBP60g* using the uORFs_*TBF1*_ strategy (Extended Data Fig. 10d), which enabled tightly controlled protein translation in response to pathogen infection^39^. As shown in Fig. 4a–c, *35S::uORFs*_*TBF1*_*-CBP60g* plants maintained basal *Pst* DC3000 resistance and pathogen-induced SA production at 28 °C. These plants also maintained substantial ETI against *Pst* DC3000(*avrPphB*) and *Pst* DC3000(*avrRps4*) at elevated temperature (Fig. 4d). Of note, *35S::uORFs*_*TBF1*_*-CBP60g* plants showed normal growth and flowering time (Fig. 4e and Extended Data Fig. 10a), demonstrating the promise of leveraging calibrated *CBP60g* expression to preserve plant immunity without detrimental growth or developmental effects.

## Discussion

We have identified *CBP60g* transcription as a major thermosensitive step in the plant immune system (Fig. 4f). Mechanistically, we observed that elevated temperature negatively affects nuclear GDAC formation and recruitment of GBPL3 and SA-relevant Mediator subunits to the *CBP60g* promoter. We identified two GDAC subpopulations in vivo*—*one sensitive to growth at 28 °C (associated with GBPL3 recruitment to the *CBP60g* promoter), whereas the other was insensitive to growth at 28 °C (associated with GBPL3 recruitment to the *NPR1* promoter). The two GDAC subpopulations could arise from different affinities for the respective promoters, access to different chromatin microenvironments, or the interacting client protein partners involved.

Recent studies have begun to implicate protein condensate formation in the environmental regulation of plant growth^10^, flowering^40^ and germination^41^. Together with these studies, our results support an emerging general concept that biomolecular condensates serve as an important regulatory node for plant sensing and/or response to external temperature and other environmental cues. CBP60g family transcription factors are widely conserved across plant lineages^12^. *35S::CBP60g*-mediated temperature resilience applies to both basal and ETI-mediated pathogen resistance, suggesting that the basic findings in this study, with further optimization, may provide a framework for broadly preserving the overall function of the plant immune system in a warming climate.

## Methods

### Plant materials

*A. thaliana* plants were grown in soil (2:1 *Arabidopsis* Mix: perlite) covered with or without standard Phifer glass mesh for 3–4 weeks at 21 °C–23 °C and 60% relative humidity under a 12 h light/12 h dark regimen (100 ± 10 µmol m^−2^ s^−1^). Accessions, mutants and transgenic lines are outlined in Supplementary Table 3. All experiments with *35S::CBP60g* were performed with line no. 17, unless otherwise specified.

Seeds of rapeseed (*Brassica napus*) cultivar Westar, tomato (*Solanum lycopersicum*) cultivar Castlemart, and tobacco (*Nicotiana tabacum*) cultivar Xanthi were grown in *Arabidopsis* Mix soil supplemented with 1 g l^−1^ of 20-20-20 general purpose fertilizer (Peters Professional). After 2 days of imbibition, plants were grown in growth chambers (20 °C/18 °C, 16 h day/8 h night for rapeseed; 23 °C/23 °C; 12 h day/12 h night for tomato and tobacco) for 4–7 weeks.

Seeds of rice (*Oryza sativa*) cultivar Nippponbare were germinated on wet filter paper in petri dishes and 4- to 5-day-old seedlings were transplanted to Redi-earth soil. Seedlings were grown at 28 °C (16 h day/8 h night) for 4–5 weeks.

### Generation of constructs and transgenic lines

To generate transgenic *Arabidopsis* harbouring *35S::uORFs*_*TBF1*_*-CBP60g*, *35S::TGA1-4myc*, or *35S::SARD1*, genomic DNA (*CBP60g*, *TGA1*) or coding sequences (*SARD1*) were amplified and ligated into pENTR D-TOPO (Invitrogen). To clone *TBF1* uORF sequence, PCR-amplified uORFs_*TBF1*_^39^ amplicon was ligated into pENTR-*AtCBP60g* using HiFi DNA Assembly (New England Biolabs). The uORFs_*TBF1*_-*CBP60g*, *TGA1* or *SARD1* construct was subcloned to pGWB517 through Gateway Cloning (Invitrogen). Plasmids carrying gene constructs were transformed into *Agrobacterium tumefaciens* GV3101, which was used for *Arabidopsis* transformation by floral dipping^42^. T1 plants were selected on half-strength Murashige and Skoog medium supplemented with hygromycin (35 mg l^−1^) and 1% sucrose. Homozygous T2 and T3 transgenic plants were analysed.

To generate *35S::ICS1* plants, the *ICS1* cDNA was amplified from RNA extracted from infected *Arabidopsis* leaves and ligated into pCR Blunt TOPO (Invitrogen). Full-length cDNA with chloroplast transit sequence was confirmed and the *35S::ICS1* construct was subcloned into pCAMBIA3301 modified to remove the GUS reporter and to include a C-terminal V5-His_6_ tag (Invitrogen) resulting in pSM200-1. pSM200-1 was transformed into *A. tumefaciens* GV3101 and used to transform *Arabidopsis eds16-1* mutant by floral dipping^42^. T1 plants were selected for glufosinolate tolerance using Finale and surviving plants were selfed and tested for presence of the insert using PCR. Homozygous T4 transgenic plants were analysed.

To generate transgenic rapeseed harbouring *35S::AtCBP60g-myc*, the *AtCBP60g* coding sequence, amplified from *Arabidopsis* cDNA, or the corresponding genomic sequence was cloned into pGWB517 through Gateway reaction (Invitrogen). The binary vector was introduced into *A. tumefaciens* GV3101 by electroporation. *B. napus* cultivar Westar were transformed using *Agrobacterium*-mediated method^43^. After 7-day explant-recovery period following co-cultivation on MS medium with benzyladenine (3 mg l^−1^), and timentin antibiotic (300 mg l^−1^) to eliminate *Agrobacterium*, putative transformants with roots (T_0_) were transferred to soil. Genomic DNA was extracted from young leaves using cetyltrimethylammonium bromide method and used for PCR detection of transgene. Two primer pairs for the hygromycin phosphotransferase (*HPT*) and *AtCBP60g* genes in the transgene were used to assess transformation. About ten T_0_ transgenic lines were used to produce T_1_ transgenic plants by self-pollination. RT–qPCR was used to screen for independent T1 transgenics that robustly expressed the *AtCBP60g* transcript. *35S::AtCBP60g* line no. 1-12 was derived from the cDNA construct, whereas *35S::AtCBP60g* line no. 2-11 was derived from the genomic DNA construct.

PCR primers are listed in Supplementary Table 4 and sequences were confirmed by Sanger sequencing.

### *Agrobacterium*-mediated transient expression in rapeseed and tobacco

For transient expression in rapeseed, *Agrobacterium* GV3101 harbouring *35S::mRFP-4myc* or *35S::AtCBP60g-4myc* was grown in Luria-Bertani (LB) medium, resuspended in infiltration buffer (10 mM MES (pH 5.7), 10 mM MgCl_2_ and 500 µM acetosyringone) at OD_600_ = 0.1, and infiltrated to the first and second true leaves of rapeseed plants using a needleless syringe. For transient expression in tobacco (*N. tabacum*), *Agrobacterium* GV3101 harbouring *35S::eGFP-GBPL3* or *35S::mRFP-MED15-flag* was grown in LB medium, resuspended in the same infiltration buffer at OD_600_ = 0.1, and infiltrated to fully expanded leaves of tobacco plants using a needleless syringe. Agroinfiltrated rapeseed or tobacco plants were incubated for 2–3 days at 21–23 °C before experiments.

### Temperature conditions

Based on previous studies^15,44–46^, *Arabidopsis* plants were acclimated at 23 °C (ambient) or 28 °C (elevated) for 24 h before chemical treatment and/or 48 h before pathogen infiltration, unless otherwise specified. Four- to five-week-old rapeseed plants were incubated at ambient (23 °C) or elevated temperatures (28 °C) for 48 h before pathogen infiltration or chemical treatments. Four- to five-week-old tomato plants were incubated at ambient (23 °C) or elevated temperatures (28 °C–32 °C) for 48 h before chemical treatments. Five-week-old rice plants were incubated at ambient (28 °C) or elevated temperatures (35 °C) before chemical treatments. Four- to seven-week-old tobacco plants were incubated at ambient (23 °C) or elevated temperatures (28 °C) for 48 h before chemical treatments. All plants were grown with a 12 h day/12 h night cycle, except for rice and rapeseed plants, which were grown with a 16 h day/8 h night cycle.

### Growth and developmental phenotyping

For growth biomass measurements, aboveground parts of 4- or 6-week-old pre-flowering plants were weighed, and representative plants were photographed. For flowering time measurements, the first instance of floral appearance for each individual plant was recorded.

### BTH and flg22 treatments

*Arabidopsis* plants were infiltrated or sprayed with mock (0.1% DMSO), benzo(1,2,3)thiadiazole-7-carbothioic acid-S-methyl ester (BTH; Chem Service, 100 µM, 0.1% DMSO) or flg22 peptide (EZBiolab, 200 nM in 0.1% DMSO). For tomato or rapeseed, 50 µM (rapeseed) or 100 µM (tomato) of BTH solution (0.02% Silwet L-77 and 0.1% DMSO) or solvent control was sprayed. Plants were further incubated for 24 h. For rice, 200 µM of BTH solution (0.1% Silwet L-77 and 0.1% DMSO) or solvent control was sprayed. Rice plants were further incubated for 24 h and their 4th leaves were used for analyses.

### Basal disease-resistance assay

Plants were infiltrated with 0.5 to 1.5 × 10^6^ CFU ml^−1^ (OD_600_ = 0.0005; for *Arabidopsis*) or 0.5 to 1.5 × 10^5^ CFU ml^−1^ (OD_600_ = 0.00005; for rapeseed) of *Pst* DC3000, 0.5 to 1.5 × 10^8^ CFU ml^−1^ of *Pst* DC3000 *ΔhrcC* (OD_600_ = 0.05; for *Arabidopsis*) or 0.5 to 1.5 × 10^6^ CFU ml^−1^ of *P. syringae* (*Ps*) pv. *tabaci* 11528 (for tobacco) as described previously^15^. Plants were returned to growth chambers at the appropriate temperature and 60% relative humidity. Bacterial levels were measured as previously described^15,47^.

### ETI assay

Plants were dipped in 0.5 to 1.5 × 10^8^ CFU ml^−1^ of *Pst* DC3000(*avrPphB*)^48^ and *Pst* DC3000(*avrRps4*)^49^ (OD_600_ = 0.05) as described previously^24,47^. Plants were left at room temperature for 1 h with a cover dome to maintain high humidity and then returned to the growth chamber without covering at either 23 °C or 28 °C (60% relative humidity). Bacterial growth was measured as described in the previous section.

### Gene expression analyses

RNA extraction and quantitative PCR analyses were performed as described previously^15^. Twenty to sixty milligrams of fresh leaf tissues were flash-frozen in liquid nitrogen and ground using a TissueLyser (Qiagen). Plant RNA was extracted using a Qiagen Plant RNeasy Mini Kit following the manufacturer’s protocol, including on-column DNase I digestion. cDNA was synthesized by adding 100–300 ng of RNA to a solution of oligo-dT primers, dNTPs and M-MLV reverse transcriptase (Invitrogen). Approximately 1.5 ng of cDNA was mixed with the appropriate primers (Supplementary Table 4) and SYBR master mix (Applied Biosystems). Quantitative PCR (qPCR) was run on a 7500 Fast Real-Time PCR system or QuantStudio 3 Real-Time PCR system (Applied Biosystems), with 2–4 biological replicates (and 3 technical replicates for each biological replicate) per experimental treatment. StepOnePlus (Applied Biosystems) was used for data acquisition and analysis. Gene expression values were calculated as described previously^15^ with the following internal controls: *PP2AA3* (*Arabidopsis*), *SlARD2* (tomato), *OsUBC* (rice), *NtAct* (tobacco) and *BnaGDI1* (rapeseed). RT–qPCR primer sequences are listed in Supplementary Table 4.

### Transcriptome analyses

For RNA-seq in Fig. 1, *Arabidopsis* Col-0 plants were inoculated with mock (0.25 mM MgCl_2_) or *Pst* DC3000 suspension, and then incubated at 23 °C or 30 °C for 24 h. For RNA-seq in Fig. 3, *Arabidopsis* Col-0 and *35S::CBP60g* were inoculated with *Pst* DC3000 suspension, and then incubated at 23 °C or 28 °C for 24 h. Total RNA was extracted as described above. RNA samples for each treatment were checked for quality and cDNA libraries were prepared, as described previously^15^. All 12 libraries per experiment were pooled in equimolar amounts for multiplexed sequencing. Pools were quantified using the Kapa Biosystems Illumina Library Quantification qPCR kit, and loaded on one lane (Fig. 1) or two lanes (Fig. 3) of Illumina HiSeq 4000 Rapid Run flow cells. RNA-seq and analyses were performed as described previously^15^. For Fig. 1, results were filtered for *Pst* DC3000-induced or -repressed genes using a pathogen/mock fold change > 2. Temperature-downregulated, neutral and upregulated target genes were analysed for Gene Ontology (GO) enrichment using the Database for Annotation, Visualization and Integrated Discovery^50^ (DAVID; https://david.ncifcrf.gov/). For Fig. 3, results were further filtered for genes with RPKM values above 1 and 23 °C/28 °C RPKM ratios with at least twofold change. Filtered genes were grouped into four clusters. Cluster 1 had genes more downregulated at 28 °C in Col-0 (that is, Col/*35S::CBP60g* ratios of 23 °C/28 °C RPKM values > 2). Cluster 2 had genes more upregulated at 28 °C in Col-0 (that is, Col/*35S::CBP60g* ratios of 23 °C/28 °C RPKM values < 0.5). Cluster 3 had genes similarly downregulated, whereas cluster 4 had genes similarly upregulated in Col-0 and *35S::CBP60g*, respectively (that is, Col/*35S::CBP60g* ratios of 23 °C/28 °C RPKM values between 2 and 0.5). GO enrichment analyses were also conducted using DAVID^50^.

### Hormone profiling

Plant hormones were extracted and quantified using a previously described protocol^15^, with minor modifications. Methanolic extraction was performed with abscisic acid (ABA)-d_6_, SA-d_4_ or SA-^13^C_6_ as an internal control. Filtered extracts were analysed using an Acquity Ultra Performance Liquid Chromatography system coupled to a Quattro Premier XE MS/MS (Waters) or a 1260 infinity High Performance Liquid Chromatography system coupled to a 6460 Triple Quadrupole mass spectrometer (Agilent). Column temperature was set at 40 °C with a 0.4 ml min^−1^ flow rate and a gradient of mobile phases water + 0.1% formic acid (A) and methanol (B) was used as follows: 0–0.5 min 2% B; 0.5–3 min 70% B; 3.5–4.5 100% B; 4.51–6 min 2% B; followed by additional 1 min for equilibration. Eluted analytes were introduced into Agilent jet stream electro spray ionization ion source and analysed in negative ion mode with delta EMV (–) of 200. The following parameters were used for the mass spectrometer source: gas temperature, 300 °C; gas flow, 5  min^−1^; nebulizer, 45 psi; sheath gas temperature, 250 °C; sheath gas flow, 11 l min^−1^; capillary voltage, 3,500 V; nozzle voltage, 500 V. The following parameters were used for data acquisition in multiple reaction monitoring (MRM) mode: dwell time, 50 ms; cell accelerator voltage, 4 V; fragmentor voltage, 90 V and collision energy, 16 V for SA and SA-d4; fragmentor voltage, 130 V and collision energy, 9 V for ABA-d6. The following MRM transitions were monitored: SA (*m/z* 137→93), SA-d_4_ (*m/z* 141→97) and ABA-d_6_ (*m/z* 269.1→159.1). Peak selection and integration of acquired MRM data files was done using QuanLynx v4.1 software (Waters) or Quantitative Analysis (for QQQ) program in MassHunter software (Agilent). Analyte levels were calculated as previously indicated^15^.

### Nuclear–cytoplasmic fractionation

Approximately 0.1–0.2 g of ground plant tissues (pre-frozen, stored at −80 °C for less than 1 week) were dissolved in nuclei isolation buffer (20 mM Tris-Cl pH 7.5, 25% glycerol, 20 mM KCl, 2.5 mM MgCl_2_, 2 mM EDTA, 250 mM sucrose, 1× protease inhibitor cocktail (Roche)) on ice (NPR1–YFP protein analysis) or at 23 °C or 28 °C (GBPL3 protein analysis). After removing debris by filtering with two layers of Miracloth (Millipore), collected extracts were centrifuged at 1,000*g* for 10 min at cold room or at 23 °C or 28 °C using a temperature-controlled centrifuge. Supernatants were collected as the cytosolic fraction and pellets were suspended in nuclei washing buffer (nuclei isolation buffer supplemented with 0.1 % Triton X-100) (Sigma-Aldrich) by gentle tapping and centrifuged at 1,000*g* for 10 min at 4 °C. After washing twice, pellets were resuspended in nuclei isolation buffer and collected as nuclear fractions, which were further used for analysis.

### Chromatin immunoprecipitation

ChIP was performed as previously reported^51^, with some modifications. Collected fresh leaf tissues were fixed (1% formaldehyde in 1× phosphate buffered saline (PBS)) by vacuum infiltration and incubated for 10–15 min to crosslink at room temperature. After quenching the remaining fixation solution with 125 mM glycine solution for 5 min, plant tissues were flash-frozen in liquid nitrogen and ground by mortar and pestle. Six-hundred milligrams of ground powder were dissolved in 2 ml of nuclei isolation buffer and crude extracts were filtered with two layers of Miracloth (Millipore). To collect nuclei, the filtrate was centrifuged at 10,000*g* at 4 °C for 5 min and the pellet was suspended in 75 µl of nuclei lysis buffer (50 mM Tris pH 8.0, 10 mM EDTA pH 8.0, 1% SDS). After 30 min incubation on ice, 625 µl of ChIP dilution buffer (16.7 mM Tris pH 8.0, 167 mM NaCl, 1.2 mM EDTA, 1.1% Triton X-100, 0.01% SDS) were added and the samples were sonicated for 1 min in the cold room using Sonic Dismembrator (Thermo Fisher) or 5–6 min using Bioruptor (Diagenode). After adding 200 µl of ChIP dilution buffer and 100 µl of 10% Triton X-100, samples were spun at full speed for 5 min to remove debris. For pre-clearing, samples were incubated with 25 µl of magnetic protein A or G beads (Thermo Fisher) for 2 h in the cold room. Twenty microlitres of samples were removed as 2% input samples. To capture the DNA–protein complex, antibodies (Supplementary Table 5) were used for immunoprecipitation and samples were incubated (with rotation) overnight in the cold room using a tube rotator. After washing, DNA samples were recovered using elution buffer and incubated overnight at 65 °C to remove crosslinking. DNA samples were collected and purified using a QIAquick PCR Purification Kit (Qiagen). ChIP–qPCR was performed as described in ‘Gene expression analyses’. ChIP–qPCR primer sequences are listed in Supplementary Table 4.

### Immunoblot

Ground plant tissues (0.2 g per 1 ml LDS buffer (Genscript)) or fractionated protein samples (1:1 v/v) were mixed with 2× LDS buffer in the presence or absence of 2-mercaptoethanol (Sigma-Aldrich) and boiled at 70 °C for 5 min. After removing debris by centrifugation, protein samples were resolved using SDS–PAGE (SurePAGE, Genscript) and transferred to PDVF membrane (Millipore) using a wet transfer system (Bio-Rad; transfer buffer from Thermo Scientific) for further analysis. Transferred blot was incubated in PBS-T (1× PBS, 0.05 % Tween-20) supplemented with 5% non-fat dried milk for 1h and relevant proteins were detected using specific antibodies. Chemiluminescence from blots was generated after adding Supersignal West dura or West femto substrate (Thermo Scientific) and detected by a ChemiDoc MP imaging system (Bio-Rad) or iBright CL 1500 (Thermo Scientific). Relative protein quantification was performed using iBright CL 1500 (Thermo Scientific) and FIJI/ImageJ software (win64 1.52i version). Experimental conditions for antibodies are in Supplementary Table 5.

### Confocal laser scanning microscopy and image analysis of *Arabidopsis* and tobacco cells

Images were acquired with the Zeiss confocal laser scanning microscopy 880 system and Zen black software (Carl Zeiss). Pre-treated leaves of 4- to 5-week-old plants (*35S::eGFP-GBPL3*) were imaged with an inverted Zeiss 880 single point scanning confocal attached to a fully motorized Zeiss Axio Observer microscope base, with Marzhauser linearly encoded stage and a 63× NA 1.4 oil plan apochromatic oil immersion objective lens. Images were acquired by frame (line) scanning unidirectionally at 0.24 microseconds using the galvanometer-based imaging mode, with a voxel size of 0.22 µm × 0.22 µm × 1 µm and an area size of 224.92 µm × 224.92 µm × 1 µm µm in Zeiss Zen Black Acquisition software and saved as CZI files. eGFP and chlorophyll was excited at 488 nm excitation laser from argon laser source and detected at 490–526 or 653–683 nm, respectively. Equal acquisition conditions (for example, excitation laser source intensity, range of acquired emission light range and exposure condition) were used for every image in each experiment. To maintain appropriate temperature during experiments, a portable temperature chamber and temperature-controlled specimen chamber of confocal microscope were used. To analyse images, FIJI/ImageJ software (Windows 64 1.52i version) was used.

### Prediction of intrinsically disordered region of *A. thaliana* MED15

The *A. thaliana* MED15 protein (encoded by *At1g15780*) disordered region was calculated with the Predictor of Natural Disordered Regions online tool (http://www.pondr.com/). The MED15 amino acid sequence was obtained from The Arabidopsis Information Resource (TAIR; https://www.arabidopsis.org/).

### Experimental design and statistical analysis of dataset

Sample size and statistical analyses are described in the relevant figure legends. Sample size was determined based on previous publications with similar experiments to allow for sufficient statistical analyses. There were no statistical methods used to predetermine sample sizes. Three to four plants (biological replicates) per genotype per treatment were analysed per individual experiment. Plants of different genotypes were grown side by side in environmentally controlled growth chambers (light, temperature, humidity) to control other covariates and to minimize unexpected environmental variations. Leaf samples of similar ages were collected and assessed randomly for each genotype. Researchers were not blinded to allocation during experiments and outcome assessment. This is in part because different plant genotypes, temperatures and treatments investigated exhibit quite distinct and obvious phenotypes visually; thus, blinding was not possible in these cases. Routine practices included more than one author observing/assessing phenotypes, whenever possible. Three or more independent experiments were performed for all assays, unless specified otherwise. The following statistical analyses were employed: (1) Student’s *t*-test with Bonferroni test for significance was used for pairwise comparisons; (2) one-way analysis of variance (ANOVA) with Bartlett’s test for significance was used for multi-sample experiments with one variable; and (3) two-way ANOVA followed by Tukey’s honest significant difference test was used for multi-variable analyses. Statistical tests are described in the figure legends. Bar graphs and dot plots were generated with GraphPad Prism 9 and show the mean ± s.d. or mean ± s.e.m. and individual data points.

### Graphic design

Figs. 1a, 2f and 4f and Extended Data Fig. 7a were created in part using BioRender.com.

### Reporting summary

Further information on research design is available in the Nature Research Reporting Summary linked to this paper.

## Online content

Any methods, additional references, Nature Research reporting summaries, source data, extended data, supplementary information, acknowledgements, peer review information; details of author contributions and competing interests; and statements of data and code availability are available at 10.1038/s41586-022-04902-y.

## Supplementary information

Supplementary Information This file contains Supplementary Fig. 1 and Supplementary Tables 1–5.

Reporting Summary

Supplementary Data 1 RNA-seq reads of the mock- and *Pst* DC3000-inoculated *Arabidopsis* Col-0 plants at normal and elevated temperature.

Supplementary Data 2 *Pst* DC3000- and temperature-regulated gene clusters.

Supplementary Data 3 RNA-seq reads of the *Pst* DC3000-inoculated *Arabidopsis* Col-0 and *35S::CBP60g* plants at 23 °C and 28 °C.

Supplementary Data 4 List of genes that are differentially regulated between Col-0 and *35S::CBP60g*.

## Data Availability

RNA-seq datasets are publicly available in the Gene Expression Omnibus under accessions GSE152072 and GSE197771). Source data are provided with this paper.

## Source data

Source Data Fig. 1
Source Data Fig. 2
Source Data Fig. 3
Source Data Fig. 4
Source Data Extended Data Fig. 1
Source Data Extended Data Fig. 2
Source Data Extended Data Fig. 3
Source Data Extended Data Fig. 4
Source Data Extended Data Fig. 5
Source Data Extended Data Fig. 6
Source Data Extended Data Fig. 7
Source Data Extended Data Fig. 8
Source Data Extended Data Fig. 9
Source Data Extended Data Fig. 10
