# Supplementary material for: Increasing the resilience of plant immunity to a warming climate
Source: Nature. 2022 Jun 29;607(7918):339–44. doi: 10.1038/s41586-022-04902-y (PMC9279160; doi:10.1038/s41586-022-04902-y)
Supplement: Supplementary file 1 — This file contains Supplementary Fig. 1 and Supplementary Tables 1–5. [file 41586_2022_4902_MOESM1_ESM.pdf]

nature portfolio

https://doi.org/10.1038/s41586-022-04902-y

Supplementary information

Increasing the resilience of plant immunity to a warming climate

In the format provided by the authors and unedited

Increasing the resilience of plant immunity to a warming climate

Jong Hum Kim[1,2,3,†], Christian Danve M. Castroverde[3,4,5,†,*], Shuai Huang[6,7,8], Chao Li[9], Richard Hilleary[1,2,3], Adam Seroka[1,2,4,10], Reza Sohrabi[1,2,3,4], Diana Medina-Yerena[3], Bethany Huot[1,4], Jie Wang[10], Kinya Nomura[1,2,3], Sharon K. Marr[11], Mary C. Wildermuth[11], Tao Chen[9], John D, MacMicking[6,7,8], and Sheng Yang He[1,2,3,4,10,*]

[1]Department of Biology, Duke University, Durham, NC 27708, USA.

[2]Howard Hughes Medical Institute, Duke University, Durham, NC 27708, USA.

[3]Department of Energy Plant Research Laboratory, Michigan State University, East Lansing, MI 48824, USA.

[4]Plant Resilience Institute, Michigan State University, East Lansing, MI 48824, USA.

[5]Department of Biology, Wilfrid Laurier University, Waterloo, ON N2L 3C5, Canada.

[6]Howard Hughes Medical Institute, Yale University, West Haven, CT 06516, USA.

[7]Yale Systems Biology Institute, Yale University, West Haven, CT 06516, USA.

[8]Departments of Immunobiology and Microbial Pathogenesis, Yale University School of Medicine, New Haven, CT 06516, USA.

[9]State Key Laboratory of Agricultural Microbiology, Huazhong Agricultural University, Wuhan, Hubei Province, China.

[10]Department of Plant Biology, Michigan State University, East Lansing, MI 48824, USA.

[11]Department of Plant and Microbial Biology, University of California Berkeley, Berkeley, CA 94720, USA.

*Correspondence to: Sheng Yang He (Email: shengyang.he@duke.edu) and Christian Danve M. Castroverde (Email: dcastroverde@wlu.ca)

†Authors contributed equally to this work.

Supplementary Figure 1. Gel/Immunoblot Source Data.

Extended Data Fig.3g

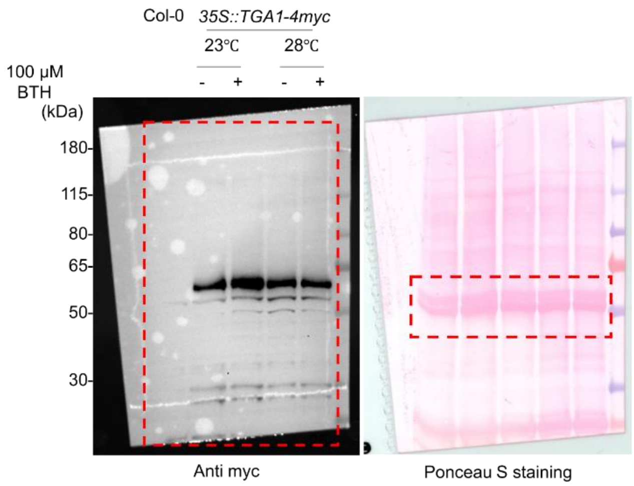

IB: anti myc (1:5000; abcam rabbit; ON; 5% skim milk)
anti mouse-HRP (1:2000; GE; 1h; 5% skim milk)

Extended Data Fig.3i

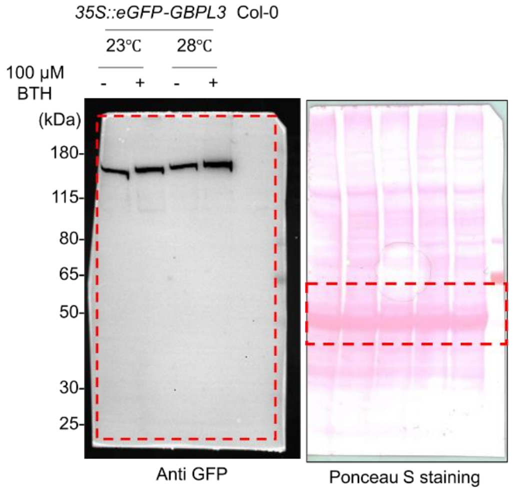

IB: anti GFP (1:2000;clontech mouse; ON; 5% skim milk)
anti mouse-HRP (1:2000; GE; 1h; 5% skim milk)

Extended Data Fig.3h

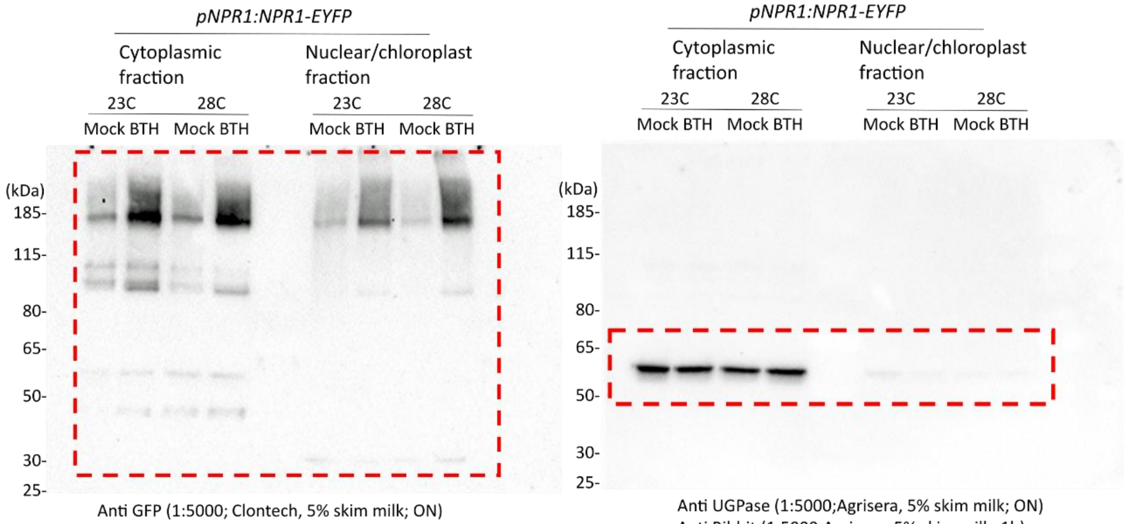

Anti GFP (1:5000; Clontech, 5% skim milk; ON)
Anti Mouse (1:5000; Cell signaling; 5% skim milk; 1h)

Anti UGPase (1:5000;Agrisera, 5% skim milk; ON)
Anti Ribbit (1:5000;Agrisera; 5% skim milk; 1h)

Extended Data Fig.3j

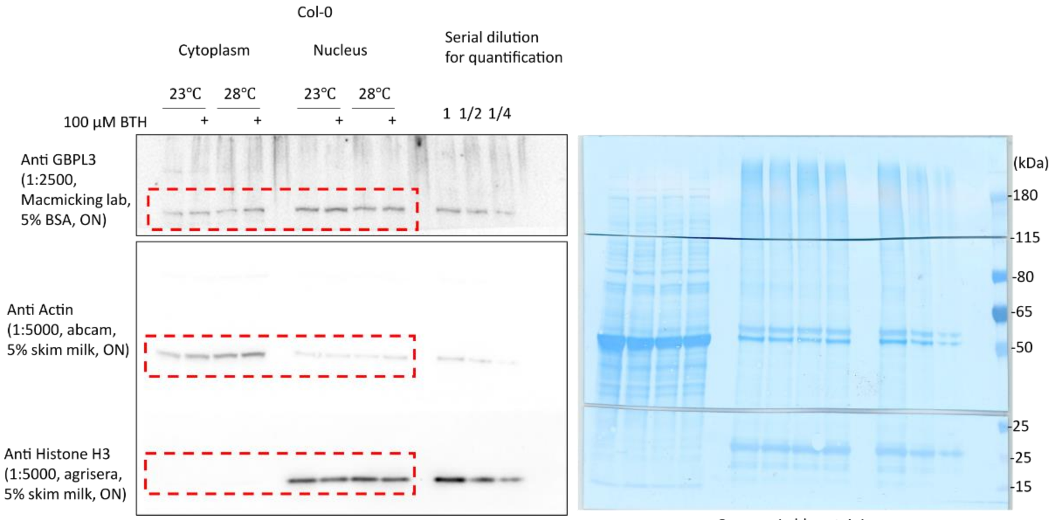

2nd Anti rabbit (1:2000, GE, 5% skim milk, 1h)

Coomassie blue staining

Extended Data Fig.3k

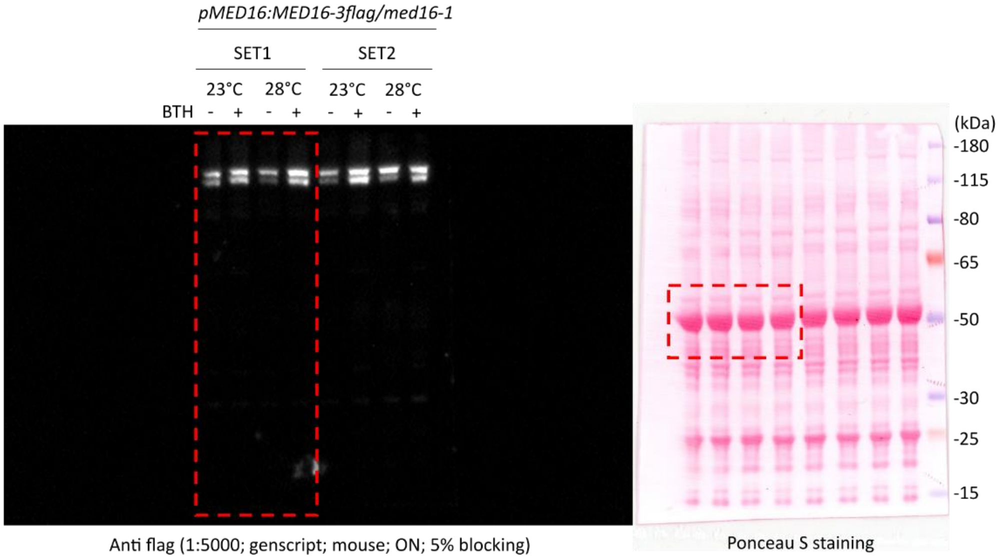

Anti flag (1:5000; genscript; mouse; ON; 5% blocking)
Anti mouse (1:5000; GE; 1hr; 5% blocking)

Extended Data Fig.3m

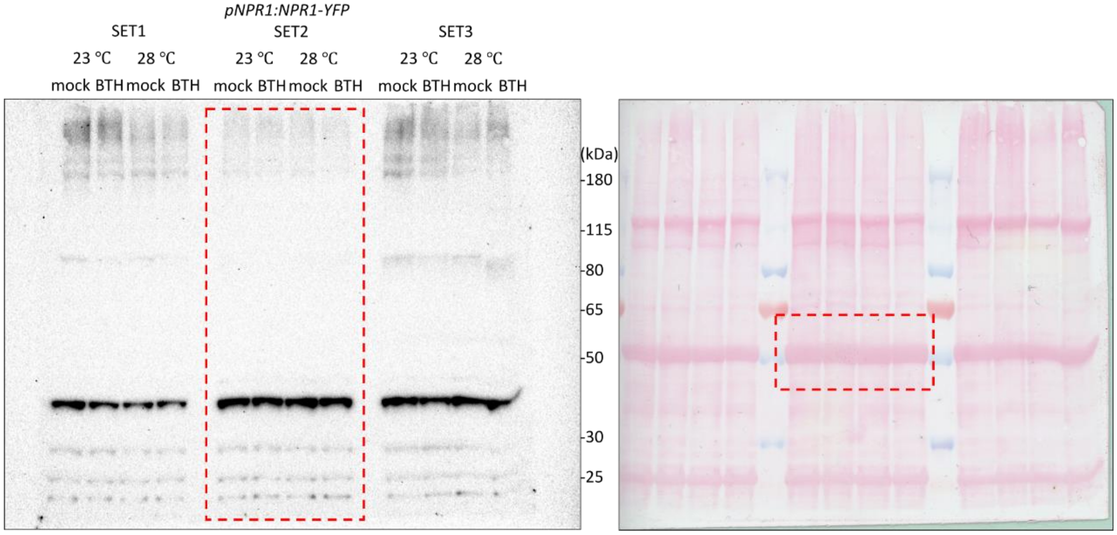

Anti Med6 (1:2000; ON; agrisera; PBS-T)
Anti rabbit (1:10000; 1h;agrisera ; 5% skim milk)

Ponceau S staining

Extended Data Fig.3o

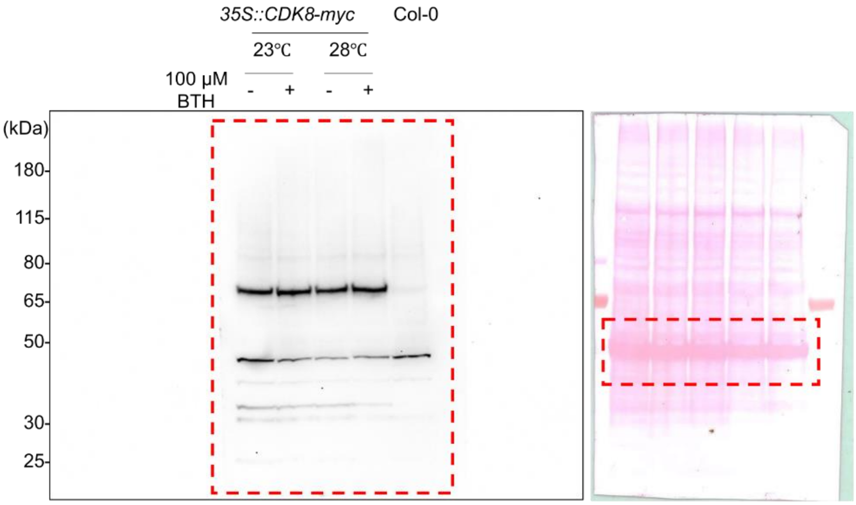

anti-myc

Ponceau S staining

IB: anti myc (1:2500;abcam rabbit; ON; 5% skim milk)
anti rabbit-HRP (1:10000; agrisera; 1h; 5% skim milk)

Supplementary Table 1. Transcriptome clustering and GO-enrichment of *Pst* DC3000-regulated genes in *Arabidopsis* plants at 23ºC (ambient) vs. 30ºC (elevated temperature).

Pathogen effect	Temperature effect	Cluster	Number of genes	Top 5 GO terms	CBP60g/ SARD1 target genes
Pst DC3000-induced	Down at elevated temperature	A	717	Defense response<br>Systemic acquired resistance<br>Response to fungus<br>Response to bacterium<br>Response to salicylic acid	140
	Similar at both temperatures	B	1236	Response to jasmonic acid<br>Response to wounding<br>Negative regulation of nucleic acid-templated transcription<br>Regulation of jasmonic acid mediated signaling pathway<br>Jasmonic acid biosynthetic process	126
	Up at elevated temperature	C	619	Response to jasmonic acid<br>Response to wounding<br>Response to high light intensity<br>Metabolic process<br>Response to karrikin	22
Pst DC3000-suppressed	Down at elevated temperature	D	845	Response to chitin<br>Protein phosphorylation<br>Response to light stimulus<br>Response to cold<br>Purine nucleobase transport	83
	Similar at both temperatures	E	1501	Circadian rhythm<br>Response to auxin	66

				Regulation of Transcription, DNA-templated<br>Xylan catabolic process<br>Auxin-activated signaling pathway	
	Up at elevated temperature	F	402	Cold acclimation<br>Response to water<br>Transcription, DNA-templated<br>Response to water Deprivation<br>Response to abscisic acid	3

Differentially expressed genes (DEGs) were categorized as either pathogen-induced or pathogen-suppressed. These DEGs were further classified as downregulated at elevated temperature, similar between the two temperatures ("Similar) and upregulated at elevated temperature. The number of genes and top 5 Gene Ontology (GO) terms based on DAVID[50]are indicated, along with how many of these genes are present in the *CBP60g* homolog *SARD1* ChIP-Seq dataset[31].

Supplementary Table 2. Transcriptome clustering and GO-enrichment of temperature-regulated genes in Col-0 and *35S::CBP60g* after *Pst* DC3000 infection.

Cluster	Definition of cluster	Number of genes	Top 5 GO terms	Example genes
1	Genes which are down-regulated in *Pst*-treated Col-0 at elevated temperature than control temperature, and expressed similarly in *Pst*-treated *35S::CBP60g* at control and elevated temperature	595	Defense response to bacterium<br><br>Cellular response to hypoxia<br><br>Response to bacterium<br><br>Response to Salicylic acid<br><br>Defense response	**SA-related:** *ICS1, PBS3, SARD1, DMR6, NIMIN1, NIMIN-2, NPR3, PR4, PR5*<br><br>**PTI-related:** *LYK5, WAK1, LYM2, BIR1, SOBIR1, RLP23, PUB23, PUB24, MKK2, MEK1, MPK4, CNGC2/10*<br><br>**ETI-related:** *ADR1, RPS4, ZAR1, NUDT7, NHL3, EDS1, PAD4, ACD6, HR3/4, TIR*<br><br>**WRKYs:** *WRKY18, 33, 38, 46, 51, 53, 62, 70*<br><br>**RLKs:** *CRK7, 10, 12, 14, 15, 24, 34*<br><br>**Camalexin/ Glucosinolate-related:** *PAD3, ESM1, MYB51*<br><br>**Oxidative stress:** *GSTF2/6/7/8,*

				PRXCB, MLO2, SAG21
2	Genes which are up-regulated in *Pst*-treated Col-0 at elevated temperature than control temperature, and expressed similarly in *Pst*-treated *35S::CBP60g* at control and elevated temperature	443	Response to water deprivation<br><br>Response to abscisic acid<br><br>Auxin-activated signaling pathway<br><br>Response to auxin<br><br>Cold acclimation	**ABA-related:** *NCED3, RAB18, NAC019, ABI1, ABF3, HB7, HB12, PIP1;4, PIP1A, PIP2A, RAP2.1, AIB, MARD1*<br><br>**Auxin-related:** *IAA1/2, AXR3, SHY2, HLS1, HAT2, SAUR20/24/63, WES1*<br><br>**Cold-related:** *CBF2, RAB18, AZI1, COR15B, COR413-PM1, COR15A, COR47, KIN1/2, LTI78*
3	Genes, which are similarly down-regulated at elevated temperature than control temperature in *Pst*-treated Col-0 and *35S::CBP60g*	810	Cellular response to hypoxia<br><br>Response to chitin<br><br>Protein autophosphorylation<br><br>Response to cold<br><br>Defense response to bacterium	**Hypoxia-related:** *OPR1, ZAT6, STY46, FBS1, DIN10, GAE1, ACBP3, ERD15, DIC2, MEE14, HSPRO2, RAV1/2, STZ, SZF1*<br><br>**Chitin-related:** *ATL6, WRKY40/22, MYBR1, MYB73/77, ERF5/6, JAZ7, MPK3*

4	Genes which are similarly up-regulated at elevated temperature than control temperature in *Pst*-treated Col-0 and *35S::CBP60g*	593	Response to wounding<br><br>Response to jasmonic acid<br><br>Oxylipin biosynthetic process<br><br>Jasmonic acid biosynthetic process<br><br>Regulation of transcription, DNA-templated	**JA-related:** *AOS, OPR3, LOX2/3/4, JMT, JAZ5/8/10, VSP1/2, TSP03/04, ADC2, DHAR1, VTC2/5, EBP, SQE3*<br><br>**Wounding-related:** *DHS1, ORA47, PAL1, JR1, ADC2, MYB29/76, GLR3.3, GLR3.4*

Supplementary Table 3. Plant materials used in this study.

Plant Material	Species	Reference
Col-0	*Arabidopsis thaliana*	Arabidopsis Biological Resource Centre (ABRC) at The Ohio State University
Ler	*A. thaliana*	ABRC
cbp60g-1	*A. thaliana*	Wang et al., 2009[28]
35S::CBP60g OE-16	*A. thaliana*	Wan et al., 2012[52]
35S::CBP60g OE-17	*A. thaliana*	Wan et al., 2012[52]
35S::uORFs$_{TBF1}$*-CBP60g* #b5, b14	*A. thaliana*	This study
35S::ICS1	*A. thaliana*	This study
35S::TGA1-4myc	*A. thaliana*	This study
35S::SARD1-4myc #b1, b2	*A. thaliana*	This study
camta2/3	*A. thaliana*	Kim et al., 2013[53]
35S::WRKY75	*A. thaliana*	Zhang et al., 2017[54]
35S::EDS1	*A. thaliana*	Feys et al., 2005[55]
35S::PAD4	*A. thaliana*	Xing et al., 2006[56]
bsmt1	*A. thaliana*	Attaran et al., 2009[57]
npr1 S11D/S15D	*A. thaliana*	Saleh et al., 2015[27]
npr3-2 npr4-2	*A. thaliana*	Ding et al., 2018[26]
pCBP60g (1788 bp; -1800 to -14 from ATG):GUS	*A. thaliana*	Wan et al., 2012[52]
npr1-6	*A. thaliana*	Huot et al., 2017[15]
NPR1pro::NPR1-EYFP	*A. thaliana*	Huot et al., 2017[15]
MED16pro::MED16-3FLAG	*A. thaliana*	Wang et al., 2015[58]
35S::PHYB^{Y276H}	*A. thaliana*	Jones et al., 2015[59]
BdELF3-OE	*A. thaliana*	Jung et al., 2020[10]
35S::CDK8-myc	*A. thaliana*	Zhu et al., 2014[60]
35S::eGFP-GBPL3	*A. thaliana*	Huang et al., 2021[11]
GBPL3 OX (*pGBPL3::GBPL3*)	*A. thaliana*	Huang et al., 2021[11]
ics1 (i.e., *sid2-2*)	*A. thaliana*	Wildermuth et al., 2001[21]
Westar	*Brassica napus*	USDA Agricultural Research Service
35S::AtCBP60g-myc	*Brassica napus*	This study
Xanthi	*Nicotiana tabacum*	USDA Agricultural Research Service
Castlemart	*Solanum lycopersicum*	Tomato Genetics Resource Center (TGRC) at UC Davis

Nipponbare	*Oryza sativa*	USDA Agricultural Research Service

Supplementary Table 4. Oligonucleotide primers used in this study.

Target genes	Sequence (5'-3')	Purpose	Reference
CBP60g (At5g26920)	caccATGAAGATTCGGAACAGCC	Cloning	This study
	TTACAAGCCTTCCCTCGGATTTC		
uORF$_{TBF1}$	CGCGGCCGCCCCCTTCACC AACAGCATCCGTTTTTATAA	Cloning	This study
	GGGCTGTTCCGAATCTTCAT CTCCGGCGAACTTTTTTTAT		
AtTGA1 (At5g65210)	CACCATGAATTCGACATCGACACAT TTTG	Cloning	This study
	CGTTGGTTCACGATGTCGAG		
SARD1 (AT1G73805)	caccATGGCAGGGAAGAGGTTATTTC A	Cloning	This study
	GAAAGGGTTTATATGATTTTGAGAC GAAGA		
PP2AA3 (At1G13320)	GGTTACAAGACAAGGTTCACTC	qRT-PCR	Huot et al., 2017[15]
	CATTCAGGACCAAACTCTTCAG		
CBP60g (At5g26920)	TCGTGGACGCCACCACAAACA	qRT-PCR	Kim et al., 2017[61]; 2020[62]
	TCAGCGTTCAGCGGCACGAG		
ICS1 (At1g74710)	ACTTACTAACCAGTCCGAAAGACG A	qRT-PCR	Huot et al., 2017[15]
	ACAACAACTCTGTCACATATACCGT		
EDS1 (At3g48090)	TCGCTTTCCAACCATCTTTCTC	qRT-PCR	This study
	CTACTCCTCACAGCCATTTCC		
PAD4 (At3g52430)	ATCTTCTCCGCCGTCATTCC	qRT-PCR	Kim et al., 2017[61]; 2020[62]
	CACGTGGCAGAAGTTGTGTG		
WRKY75 (At5g1308)	GTTCCCTAGGAGTTACTATAGGTG	qRT-PCR	This study
	TTTCGGTGGATTTCTCGATGG		
BSMT1 (At3g11480)	CTAGTCTTTGAGGGTCTTGTG	qRT-PCR	This study
	TGAGCATTGGTTCACTAACAG		
GUS	ACGGGAAAGGACTGGAAGAG	qRT-PCR	This study
	ATGGTTGCGGATTTCCTTGG		
SlPR1b (Solyc09g00701)	CTTGCGGTTCATAACGATGC	qRT-PCR	Kusajima et al., 2017[63]
	TAGTTTTGTGCTCGGGATGC		
SlARD2 (Solyc01g104170)	TGTTCATCAGTGTGCTAGTG	qRT-PCR	Pombo et al., 2017[64]
	GCTGTCCTTCCTTCTGAATC		
OsPR1b (*Os01g0382000*)	ACGCCTTCACGGTCCATAC	qRT-PCR	Fang et al., 2015[65]
	AAACAGAAAGAAACAGAGGGAGT AC		
OsUBC (Os02g0634800)	CCGTTTGTAGAGCCATAATTGCA	qRT-PCR	Jain et al., 2006[66]
	AGGTTGCCTGAGTCACAGTTAAGTG		

BnaPR1	CATCCCTCGAAAGCTCAAGAC<br>CCACTGCACGGGACCTAC	qRT-PCR	Sasek et al., 2012[67]
BnaICS1	CAAACTCATCATCTTCCCTC<br>AGCGTGACTTACTAACCAG	qRT-PCR	Sasek et al., 2012[67]
BnaGDI1	GAGTCCCTTGCTCGTTTCC<br>TGGCAGTCTCTCCCTCAGAT	qRT-PCR	Yang et al., 2014[68]
NtPR1	CAATTAGTATGGACTTTCG<br>GCAGATTGTAACCTCGTA	qRT-PCR	Künstler et al., 2020[69]
NtActin	GCCGTCCTTAGCAGCAGT<br>ACAAGCAACCCTTCCACC	qRT-PCR	Künstler et al., 2020[69]
hygromycin phosphotransferase (HPT)	ACACTACATGGCGTGATTTCAT<br>TCCACTATCGGCGAGTACTTCT	Genotyping PCR	This study
AtCBP60g-myc	CGGTTAGGTGCTAAGTTGA<br>TCACCGTCGAGTCCGTTCAA)	Genotyping PCR	This study
TA3 retrotransposon	CTGCGTGGAAGTCTGTCAAA<br>CTATGCCACAGGGCAGTTTT	ChIP-qPCR	Yamaguchi et al., 2014[51]
CBP60g as1 (-1.1 kb, P1)	GGGGTCAATGTACTTCTAGTTGA<br>AAACTAGCCAAAACAGCCGT	ChIP-qPCR	This study
CBP60g coding sequence (P2)	GAAGATTCGGAACAGCCCTA<br>TCCGAAAATAAACCGGAAAA	ChIP-qPCR	This study
SARD1-P1	GGAACCGTCCATTTGTCAAC<br>TTCGAAGAACGACAAAGGAAA	ChIP-qPCR	Huang et al., 2021[11]
TZF1 coding sequence (*At3g55980*)	GCAGTGGACCAAAGAGCAAT<br>CAAGATCATCACAAGCAGCGA	ChIP-qPCR	This study
NPR1-P1	AATGTAAACCGTGGGACGAG<br>TAAGAATCGGCGAATCCATC	ChIP-qPCR	Huang et al., 2021[11]

Supplementary Table 5. Antibodies used in this study.

Antibody Name	Dilution Used	Purpose	Reference
Anti-GFP	1:250	ChIP	Cat. No. ab290, Abcam
Anti-GFP	1:5000	Western blotting (primary antibody)	Cat. No. 632381, Clontech
Anti-myc	1:200, 1:5000	ChIP, Western blotting (primary antibody)	Cat. No. ab9106, Abcam
Anti-FLAG	1:200	ChIP	Cat. No. A00170, Genscript
Anti-FLAG	1:2500	Western blotting (primary antibody)	Cat. No. A00187, Genscript
Anti-GBPL3	1:2500	Western blotting (primary antibody)	Huang et al., 2021[11]
Anti-Actin	1:5000	Western blotting (primary antibody)	Cat. No. ab197345, Abcam
Anti-H3	1:5000	Western blotting (primary antibody)	Cat. No. AS10 710, Agrisera
Anti-MED6	1:100, 1:1000	ChIP, Western blotting (primary antibody)	Cat. No. AS14 2802, Agrisera
Anti-RNA polymerase II	1:250	ChIP	Cat. No. ab5131, Abcam
Anti-UGPase	1:5000	Western blotting (primary antibody)	Cat. No. AS05 086, Agrisera
Anti-Rabbit-HRP	1:5000	Western blotting (secondary antibody)	Cat. No. AS09 602 Agrisera
Anti-Mouse-HRP	1:2000 - 5000	Western blotting (secondary antibody)	Cat. No. NA931 Cytiva
Anti-Mouse-HRP	1:2000 - 5000	Western blotting (secondary antibody)	Cat. No. 7076S Cell Signaling

Supplementary Data 1. RNA-Seq reads of the mock (0.25 mM $MgCl_2$)- and *Pst* DC3000-inoculated *Arabidopsis* Col-0 plants at normal and elevated temperature. (separate file)

Supplementary Data 2. *Pst* DC3000- and temperature-regulated gene clusters. (separate file)

Supplementary Data 3. RNA-Seq reads of the *Pst* DC3000-inoculated *Arabidopsis* Col-0 and *35S::CBP60g* plants at 23°C and 28°C. (separate file)

Supplementary Data 4. List of genes that are differentially regulated between Col-0 and *35S::CBP60g*. (separate file)
