# Supplementary material for: Increasing the resilience of plant immunity to a warming climate
Source: Nature. 2022 Jun 29;607(7918):339–44. doi: 10.1038/s41586-022-04902-y (PMC9279160; doi:10.1038/s41586-022-04902-y)
Supplement: Supplementary file 2 — Reporting Summary [file 41586_2022_4902_MOESM2_ESM.pdf]

nature portfolio

Corresponding author(s): Sheng Yang He

Last updated by author(s): May 15, 2022

Reporting Summary

Nature Portfolio wishes to improve the reproducibility of the work that we publish. This form provides structure for consistency and transparency in reporting. For further information on Nature Portfolio policies, see our Editorial Policies and the Editorial Policy Checklist.

Statistics

For all statistical analyses, confirm that the following items are present in the figure legend, table legend, main text, or Methods section.

n/a	Confirmed	
☐	☒	The exact sample size (*n*) for each experimental group/condition, given as a discrete number and unit of measurement
☐	☒	A statement on whether measurements were taken from distinct samples or whether the same sample was measured repeatedly
☐	☒	The statistical test(s) used AND whether they are one- or two-sided<br>*Only common tests should be described solely by name; describe more complex techniques in the Methods section.*
☒	☐	A description of all covariates tested
☐	☒	A description of any assumptions or corrections, such as tests of normality and adjustment for multiple comparisons
☐	☒	A full description of the statistical parameters including central tendency (e.g. means) or other basic estimates (e.g. regression coefficient) AND variation (e.g. standard deviation) or associated estimates of uncertainty (e.g. confidence intervals)
☐	☒	For null hypothesis testing, the test statistic (e.g. *F*, *t*, *r*) with confidence intervals, effect sizes, degrees of freedom and *P* value noted<br>*Give P values as exact values whenever suitable.*
☒	☐	For Bayesian analysis, information on the choice of priors and Markov chain Monte Carlo settings
☒	☐	For hierarchical and complex designs, identification of the appropriate level for tests and full reporting of outcomes
☒	☐	Estimates of effect sizes (e.g. Cohen's *d*, Pearson's *r*), indicating how they were calculated

Our web collection on statistics for biologists contains articles on many of the points above.

Software and code

Policy information about availability of computer code

Data collection	Gene expression and ChIP (qPCR): Thermo Fisher QuantStudio 3 system, Thermo Fisher 7500 Fast Real-Time PCR system, Thermo Fisher StepOnePlus™ Software<br>Hormone quantification: Waters MassLynx and Thermo Fisher Masshunter system<br>Western blots: Bio Rad ChemiDoc XRS+ and Thermo Fisher iBright 1500<br>Confocal microscopy: Carl-zeiss LSM 880 with Airyscan, and Zen black software<br>Photographing of plant: Nikon D5600 digital camera
Data analysis	Statistics and graph production: GraphPad Prism 9 software<br>RNASeq: Trimmomatic version 0.32, STAR version 2.5.2b, featureCounts version 1.22.3, TMM/voom, Database for Annotation, Visualization and Integrated Discovery software<br>Gene expression and ChIP (qPCR): StepOnePlus™ software<br>Hormone quantification: Waters MassLynx and Thermo Fisher Masshunter software<br>Confocal microscopy: Carl-zeiss Zen black software and FIJI/imageJ win64 1.52i software<br>Protein quantification: FIJI/imageJ win64 1.52i software, Thermo Fisher iBright 1500 system

For manuscripts utilizing custom algorithms or software that are central to the research but not yet described in published literature, software must be made available to editors and reviewers. We strongly encourage code deposition in a community repository (e.g. GitHub). See the Nature Portfolio guidelines for submitting code & software for further information.

Data

Policy information about availability of data

All manuscripts must include a data availability statement. This statement should provide the following information, where applicable:

- Accession codes, unique identifiers, or web links for publicly available datasets
- A description of any restrictions on data availability
- For clinical datasets or third party data, please ensure that the statement adheres to our policy

Data needed to evaluate this paper is available in the main text and Supplementary Information. RNA-Seq datasets are publicly available in the Gene Expression Omnibus (GSE152072; GSE197771). Uncropped gel and blot source data are provided in Supplementary Fig. 1. Source data (with statistical analyses) for Figs. 1-4 and Extended Data Figs. 1-10 are provided with this paper. Gene and protein sequence data were obtained from The Arabidopsis Information Resource (TAIR, https://www.arabidopsis.org/).

Field-specific reporting

Please select the one below that is the best fit for your research. If you are not sure, read the appropriate sections before making your selection.

☒ Life sciences ☐ Behavioural & social sciences ☐ Ecological, evolutionary & environmental sciences

For a reference copy of the document with all sections, see nature.com/documents/nr-reporting-summary-flat.pdf

Life sciences study design

All studies must disclose on these points even when the disclosure is negative.

Sample size	Sample size and statistical analyses are described in the relevant Figure legends. Sample size was determined based on previous publications with similar experiments to allow for sufficient statistical analyses. These are consistent with the literature, e.g. Huot et al. 2017 Nature Commun 8:1808; Chen et al., 2020 Nature 580:653-657; Yuan et al., 2021 Nature 592:105-109. There were no statistical methods used to predetermine sample sizes.
Data exclusions	No data that pass quality control were excluded from statistical analysis.
Replication	The number of independent replication for each experiment is described in the relevant figure legends. Two or more independent experiments were performed for all assays. Results were ensured to be reproducible in all repeats with the same trend.
Randomization	Plants of different genotypes were grown side by side in environmentally controlled growth chambers (light, temperature, humidity) to control other covariates and to minimize unexpected environmental variations. Leaf samples of similar ages were collected and assessed randomly for each genotype.
Blinding	Researchers were not blinded to allocation during experiments and outcome assessment. This is in part because different plant genotypes, temperatures and treatments investigated exhibit quite distinct and obvious phenotypes visually; thus, blinding was not possible in these cases. Routine practices included more than one author observing/assessing phenotypes, whenever possible.

Reporting for specific materials, systems and methods

We require information from authors about some types of materials, experimental systems and methods used in many studies. Here, indicate whether each material, system or method listed is relevant to your study. If you are not sure if a list item applies to your research, read the appropriate section before selecting a response.

Materials & experimental systems

n/a	Involved in the study	
☐	☒	Antibodies
☒	☐	Eukaryotic cell lines
☒	☐	Palaeontology and archaeology
☒	☐	Animals and other organisms
☒	☐	Human research participants
☒	☐	Clinical data
☒	☐	Dual use research of concern

Methods

n/a	Involved in the study	
☒	☐	ChIP-seq
☒	☐	Flow cytometry
☒	☐	MRI-based neuroimaging

Antibodies

Antibodies used	The anti-GBPL3 antibody was provided by Shuai Huang and John D. MacMicking (Yale University). Detailed description of this antibody is in the Huang et al., 2021 Nature 594:424-429. Commercially available antibodies are enlisted in Supplementary Table 5.

Validation

All antibodies used in this study are commercially available, and were validated according to manufacturer's specifications.
anti GBPL3 antibody: Huang et al., 2021 Nature 594:424-429.
anti-GFP antibody (Cat. No. ab290, Abcam): https://www.abcam.com/gfp-antibody-ab290.html
anti-GFP antibody (Cat. No. 632381, Clontech): https://www.takarabio.com/documents/Certificate%20of%20Analysis/632380/632380-632381-070313.pdf
anti-myc antibody (Cat. No. ab9106, Abcam): https://www.abcam.com/myc-tag-antibody-ab9106.html
anti-flag antibody (Cat. No. A00170, Genscript): https://www.genscript.com/antibody/A00170-DYKDDDDK_tag_Antibody_pAb_Rabbit.html
anti-flag antibody (Cat. No. A00187, Genscript): https://www.genscript.com/antibody/A00187-THE_DYKDDDDK_Tag_Antibody_mAb_Mouse.html
anti-actin antibody (Cat. No. ab197345, Abcam): https://www.abcam.com/actin-antibody-ab197345.html
anti-Histone H3 antibody (Cat. No. AS10 710, Agrisera): https://www.agrisera.com/en/artiklar/h3-histone-h3.html
anti-MED6 antibody (Cat. No. AS14 2802, Agrisera): Bäckström S, Elfving N, Nilsson R, Wingsle G, Björklund S. Purification of a plant mediator from Arabidopsis thaliana identifies PFT1 as the Med25 subunit. Mol Cell. 2007 Jun 8;26(5):717-29.
anti-RNA polymerase II antibody (Cat. No. ab5131, Abcam): https://www.abcam.com/rna-polymerase-ii-ctd-repeat-ysptsps-phospho-s5-antibody-ab5131.html
anti-UGPase antibody (Cat. No. AS05 086, Agrisera): https://www.agrisera.com/en/artiklar/ugpase-udp-glucose-pyrophosphorylase-marker-of-cytoplasm.html
anti-rabbit antibody (Cat. No. AS09 602, Agrisera): https://www.agrisera.com/en/artiklar/goat-anti-rabbit-igg-hl.html
anti-mouse antibody (Cat. No. NA931, Cytiva): https://www.cytivalifesciences.com/en/us/shop/protein-analysis/blotting-and-detection/blotting-standards-and-reagents/amersham-ecl-hrp-conjugated-antibodies-p-06260
anti-mouse antibody (Cat. No. 7076S, Cell Signaling): https://www.cellsignal.com/products/secondary-antibodies/anti-mouse-igg-hrp-linked-antibody/7076
